# ATM–ESCO2–SMC3 axis promotes 53BP1 recruitment in response to DNA damage and safeguards genome integrity by stabilizing cohesin complex

**DOI:** 10.1093/nar/gkad533

**Published:** 2023-06-28

**Authors:** Jianfeng Fu, Siru Zhou, Huilin Xu, Liming Liao, Hui Shen, Peng Du, Xiaofeng Zheng

**Affiliations:** State Key Laboratory of Protein and Plant Gene Research, School of Life Sciences, Peking University, Beijing, China; Department of Biochemistry and Molecular Biology, School of Life Sciences, Peking University, Beijing, China; State Key Laboratory of Protein and Plant Gene Research, School of Life Sciences, Peking University, Beijing, China; Department of Biochemistry and Molecular Biology, School of Life Sciences, Peking University, Beijing, China; State Key Laboratory of Protein and Plant Gene Research, School of Life Sciences, Peking University, Beijing, China; Department of Biochemistry and Molecular Biology, School of Life Sciences, Peking University, Beijing, China; State Key Laboratory of Protein and Plant Gene Research, School of Life Sciences, Peking University, Beijing, China; Department of Biochemistry and Molecular Biology, School of Life Sciences, Peking University, Beijing, China; MOE Key Laboratory of Cell Proliferation and Differentiation, School of Life Sciences, Peking University, Beijing, China; Centre for Life Sciences, Peking University, Beijing 100871, China; MOE Key Laboratory of Cell Proliferation and Differentiation, School of Life Sciences, Peking University, Beijing, China; Centre for Life Sciences, Peking University, Beijing 100871, China; State Key Laboratory of Protein and Plant Gene Research, School of Life Sciences, Peking University, Beijing, China; Department of Biochemistry and Molecular Biology, School of Life Sciences, Peking University, Beijing, China

## Abstract

53BP1 is primarily known as a key regulator in DNA double-strand break (DSB) repair. However, the mechanism of DSB-triggered cohesin modification-modulated chromatin structure on the recruitment of 53BP1 remains largely elusive. Here, we identified acetyltransferase ESCO2 as a regulator for DSB-induced cohesin-dependent chromatin structure dynamics, which promotes 53BP1 recruitment. Mechanistically, in response to DNA damage, ATM phosphorylates ESCO2 S196 and T233. MDC1 recognizes phosphorylated ESCO2 and recruits ESCO2 to DSB sites. ESCO2-mediated acetylation of SMC3 stabilizes cohesin complex conformation and regulates the chromatin structure at DSB breaks, which is essential for the recruitment of 53BP1 and the formation of 53BP1 microdomains. Furthermore, depletion of ESCO2 in both colorectal cancer cells and xenografted nude mice sensitizes cancer cells to chemotherapeutic drugs. Collectively, our results reveal a molecular mechanism for the ATM–ESCO2–SMC3 axis in DSB repair and genome integrity maintenance with a vital role in chemotherapy response in colorectal cancer.

## Graphical Abstract

**Graphical Abstract:**
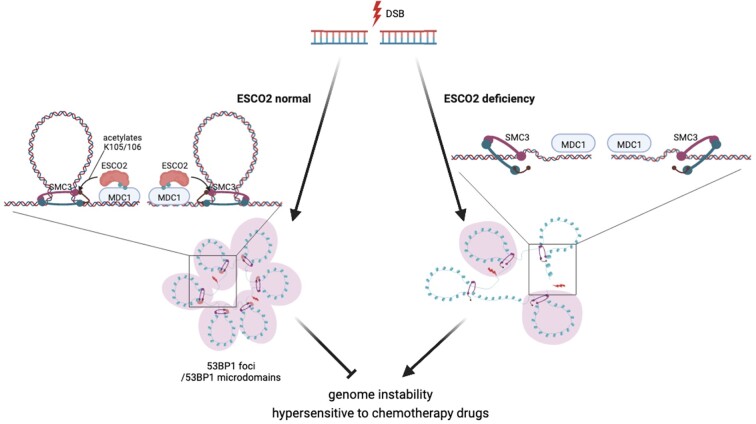

## INTRODUCTION

Double-strand breaks (DSBs) are known to be one of the most serious types of DNA lesions threatening genomic stability in mammalian cells. Failure to repair DSBs leads to loss of genetic information by mutations and chromosomal rearrangement, which contributes to the pathogenesis of cancer and diverse inherited diseases (1,2). Mammalian cells employ two major DSB repair pathways, non-homologous end joining (NHEJ) and homologous recombination (HR). NHEJ is a highly error-prone homology-independent repair mechanism that is the predominant repair pathway throughout the cell cycle, whereas HR requires a homology template, such as a sister chromatid, and occurs in the S/G2 phases of the cell cycle (3). The harmonious cooperation of the different repair pathways is critical to minimize genomic damage (4).

A DSB is detected by sensor proteins that can trigger the activation of kinases such as ATM and ATR (5). ATM phosphorylates the histone variant H2AX to generate γH2AX (6). 53BP1 is recruited to DSB sites by binding H4K20me2 and H2AK15ub in a manner that depends on the interaction of MDC1 and γH2AX (7,8). 53BP1 antagonizes the resection of DSBs in G1 by recruiting downstream RIF1, REV7, and the shieldin complex and contributes to NHEJ (8–11). 53BP1 undergoes liquid-liquid phase separation in response to DNA damage, which integrates damage detection, shielding of break sites, and checkpoint activation (12). Using 3D-SIM super-resolution microscopy, researchers have found that 53BP1 organizes DSB-flanking chromatin into circular microdomains (13). However, the regulation of 53BP1 microdomain formation and its role in the response of cancer cells to chemotherapy drugs is unclear.

Cohesin is a multiprotein, ring-shaped complex, and its canonical role is to tether sister chromatids from S phase to anaphase to prevent premature sister chromatid separation and ensure equal segregation of chromosomes (14). The cohesin complex can also regulate three dimensional chromatin organization (15) and is emerging as a key regulator in DNA damage repair by promoting homology search during recombination (16). Roberts syndrome (RBS; OMIM 268300) is a rare genetic disorder characterized by pre- and postnatal growth retardation, microcephaly, bilateral cleft lip and palate, and mesomelic symmetric limb reduction (17), and is caused by mutations in the *ESCO2* gene (18). ESCO2 acetylates the cohesin subunit SMC3 at K105/106 (19–22) and is required for the establishment of sister chromatid cohesion (23,24). However, the mechanism of ESCO2-mediated cohesin-dependent chromatin structure dynamics in DSB repair especially NHEJ repair remains to be elucidated.

In this study, we identify that acetyltransferase ESCO2 plays a role in regulating DSB repair. We show that ESCO2 is recruited to DSB sites in an ATM- and MDC1-dependent manner. Furthermore, ESCO2 promotes the formation of 53BP1 foci to DSB sites by stabilizing cohesin complex and is essential for resistance to chemotherapy in colorectal cancer cells (CRC).

## MATERIALS AND METHODS

### Cell culture and transfection

HeLa, HCT116, and RKO cells were purchased from ATCC and HEK293T was acquired from National Infrastructure of Cell Line Resource. HeLa, HCT116 and HEK293T cells were cultured in DMEM medium supplemented with 10% fetal bovine serum at 37°C with 5% CO_2_. RKO cells were cultured in RPMI 1640 medium supplemented with 10% fetal bovine serum at 37°C with 5% CO_2_. HeLa, HCT116, RKO and HEK293T cells were transfected with PEI according to the manufacturer's instructions (Polyscience).

### Plasmids

pRK5-Flag-ESCO2 and pRK5-GFP-ESCO2 plasmids were kindly provided by Huiqiang Lou at College of Biological Sciences, China Agricultural University. pX332-SMC3-EGFP plasmid was kindly provided by Xiong Ji at School of Life Sciences, Peking University. *MDC1* and truncation mutants were cloned into the pCMV-HA vector. All plasmids were verified by DNA sequencing.

### Antibodies and reagents

The following antibodies were used in our studies: mouse monoclonal anti-Flag (1:10000, F3165, Sigma-Aldrich), mouse monoclonal anti-HA (1:10 000, H9658, Sigma-Aldrich), rabbit polyclonal anti-53BP1 (IF 1:200, sc-22760, Santa Cruz Biotechnology), rabbit polyclonal anti-MDC1 (1:1000, ab11171, Abcam), rabbit polyclonal anti-BRCA1 (IF 1:100, BS6423, Bioworld), polyclonal anti-γH2AX (IF 1:200, BS4760, Bioworld), rabbit polyclonal anti-Histone H3 (1:10 000, BE3015, EASYBIO), anti-β-Tubulin (1:10 000, BE3212-10, EASYBIO), rabbit polyclonal anti-ESCO2 (1:1000, A301-689A, Bethyl), and mouse monoclonal anti-acetyl-SMC3 K105/106, Clone 21A7 (ChIP 2μg, MABE1073, Millipore). The following reagents were used in our studies: KU55933 (ATM kinase inhibitor, S1092, Selleck Chemicals), NU7441 (DNA-PK inhibitor, S2638, Selleck Chemicals), VE821 (ATR inhibitor, S8007, Selleck Chemicals), DNase I (D5025, Sigma) and bleomycin sulfate (HY-17565, MedChemExpress).

### siRNA

The MDC1 and 53BP1 siRNA oligonucleotide sequences were as follows: GUCUCCCAGAAGACAGUGAdTdT (siMDC1 #1), ACAGUUGUCCCCACAGCCCdTdT (siMDC1 #2), and AAGAUACUCCUUGCCUGAUAAdTdT (si53BP1), respectively. The control siRNA sequence was CCGAGAACACCGAGGAGAAdTdT. Cells were transfected with siRNA duplexes using the Lipo8000 Transfection Reagent (Beyotime) following the manufacturer's instructions.

### Laser microirradiation

Laser microirradiation was carried out following procedures described previously (25). HeLa cells were grown on thin glass-bottom plates and irradiated with an ultraviolet laser (16 Hz pulse, 60% laser output). Images were taken using a Dragonfly (Andor) confocal imaging system every 10 s for 30 min.

### Neutral comet assay

Neutral comet assays were performed using the Comet Assay kit (4250-050-03, Trevigen). Images were obtained using a fluorescence microscope (Olympus IX73) with a 20× objective lens. Quantification was performed using Casp Lab software v1.2.2 (University of Wroclaw, Wroclaw, Poland).

### Immunofluorescence microscopy

Cells were cultured on glass coverslips in six-well plates. Twenty-four hours later, cells were washed three times with pre-chilled PBS and then fixed with 1 ml of pre-chilled methanol for 10 min at −20°C. After washing with pre-chilled PBS three times, the cells were blocked with 1% bovine serum albumin (BSA) for 1 h, followed by incubation with primary antibodies that had been diluted in 1% BSA for 1 h at 37°C. After washing with pre-chilled PBS three times, the cells were incubated for 1 h at 37°C with secondary antibodies that had been diluted in 1% BSA and then washed with pre-chilled PBS three times. Finally, 20 μl of mounting solution was used to mount cells. Images were obtained using a confocal microscope (Zeiss LSM-710 NLO, DuoScan, and Andor dragonfly microscopy) with a 63× oil objective lens. Quantification analysis was performed using ZEN 3.1 (blue edition) (Zeiss) and ImageJ software. Super-resolution 3D-SIM imaging was carried out using a DeltaVision OMX SR (GE Healthcare). 3D image analysis was carried out using QUANTEX software (https://figshare.com/s/46fa39d1010d77f51d9c). The curvature was defined as ‘Gaussian Curvature’ and was calculated according to the method described here (https://gfx.cs.princeton.edu/pubs/Rusinkiewicz_2004_ECA/curvpaper.pdf). Curvature_Points_TH100 refers to the objects with a very high proportion of spiky curvature at a subscale >0.75 and ≤1.0.

### Clonogenic survival assay

First, 200–500 cells were seeded in six-well plates in triplicate. After 48 h, cells were cultured in medium containing a different concentration of bleomycin or oxaliplatin for 24 h and washed twice with DMEM. After 12 days, cells were washed with PBS, fixed in precooled methanol for 10 min at –20°C and stained with crystal violet (0.1% wt/vol) for 15 min. The number of clones was counted and the survival fraction was normalized to the number of untreated cells.

### Mass spectrometry

HEK293T cells were lysed in modified RIPA buffer, sonicated, and precleared with protein G beads. The supernatants were incubated with anti-Flag affinity beads at 4°C for 4 h and eluted with Flag peptide. The eluates were precipitated with TCA and subjected to mass spectrometric analysis. The MS data were aligned with the Human Reviewed Swiss-Prot database by Proteome Discoverer 2.2 software. Proteins were considered to be major hits (positive) when matching the following criteria: (i) not found in negative control group; (ii) high Protein FDR Confidence (FDR < 0.01) and (iii) peptides ≥5.

### NHEJ and HR assays

NHEJ repair assays were performed according to a protocol as previously described (26,27). WT and ESCO2 KD HCT116 cells were co-transfected with HindIII-linearized pEGFP-Pem1-Ad2 and dsRED plasmids. For the HR assay, WT and ESCO2 KD HCT116 cells were co-transfected with I-SceI, DR-GFP, and dsRED plasmids for 36 h, and the fluorescence was measured using CytoFLEX S (Beckman). The repair efficiency was determined by calculating the percentage of EGFP and dsRED double-positive cells in dsRED positive cells. The results were normalized using the WT HCT116 cells.

### Chromatin extraction assay

Cells were lysed in chromatin extraction buffer A (10 mmol/l PIPES, pH 6.8; 100 mmol/l NaCl; 300 mmol/l sucrose; 3 mmol/l MgCl_2_; 1 mmol/l EGTA; 0.2% Triton X-100) on ice for 30 min and centrifuged at 3000 *g* for 5 min. The supernatant was removed, and the cell pellets were lysed in chromatin extraction buffer B (3 mmol/l EDTA, 0.2 mmol/l EGTA, 1 mmol/l DTT) and centrifuged at 3000 *g* for 5 min. The supernatant was completely removed, and the sediment was resuspended in buffer C (50 mmol/l Tris, pH 8.0; 150 mmol/l NaCl; 1 mmol/l EDTA; 0.1% SDS; 1% Triton X-100) and denatured with 2× SDS loading buffer.

### Co-immunoprecipitation

Cells were cultured in 10-cm dishes and transfected with the indicated plasmids. After 48 h, cells were washed with 10 ml of pre-chilled PBS and lysed for 60 min in 1 ml NP-40 lysis buffer (50 mM Tris–HCl, 150 mM NaCl, 1 mM EDTA, 1% NP-40, pH 7.4) with 10 μl of protease inhibitor cocktail. Next, 1 μg of antibody or IgG was used to bind to the bait proteins for 4 h, and then incubated with 30 μl protein G for an additional 3 h. Finally, the protein G was washed with 1 ml of NP-40 lysis buffer three times and then heated for 10 min at 96°C with 30 μl 2× SDS loading buffer. Samples were analyzed by SDS-PAGE and western blotting.

### Generation of ESCO2 knockdown HCT116 and RKO cells by CRISPR-cas9 system

sgRNAs targeting different regions in the mRNA of the human *ESCO2* gene were designed and cloned into a lentiviral sgRNA vector containing the mCherry selection marker using the Golden Gate method (27). Next, cells were co-transfected with the sgRNA and Cas9 vectors. After 48 h of transfection, mCherry-positive cells were selected by FACS (MOFLO, Cytomation). Single clones were obtained after 10 days of selection. The knockout efficiency was confirmed by immunoblotting. *ESCO2* gene mutations were verified with PCR and sequencing.

### ChIP-seq and ChIP-qPCR

HCT116 cells were treated with 4-OHT (500 nM) for 4 h. For each precipitation, 1 × 10^7^ cells were crosslinked by the addition of formaldehyde directly to the growth medium to a final concentration of 1%. After 10 min, crosslinking was quenched by the addition of glycine to a final concentration of 0.25 M at room temperature. Crosslinked cells were washed with PBS, scraped, and washed again with PBS containing 1 mM EDTA, and then lysed gently on ice for 5 min using 0.5 ml of ice-cold NP-40 lysis buffer. Cell lysates were added on top of a 1.25 ml sucrose cushion (24% sucrose (wt/vol) in NP-40 lysis buffer) and centrifuged at 12 000 rpm for 10 min at 4°C to isolate the nuclei pellet. The chromatin pellet was washed twice with 1 ml PBS/1 mM EDTA, followed by centrifugation at 12 000 rpm for 1 min at 4°C. The supernatant was discarded, the tube was pre-warmed at 37°C for 2 min, and then 40 U MNase was added followed by incubation at 37°C for 15 min with rotation at 700 rpm. Subsequently, the MNase was inactivated by the addition of 20 μl 0.5 M EDTA (to a final concentration of 10 mM) and 40 μl 0.5 M EGTA (to a final concentration of 20 mM). The mixture was then placed on ice, mixed thoroughly by pipetting, and sonicated using pre-cooled Biorupter at 15–20× (30 s on/30 s off) on high position. The sonicated chromatin was spun down at 12 000 rpm for 10 min at 4°C to collect the supernatant chromatin. Next, 1–2 μg antibodies against γH2AX, 53BP1, and ac-SMC3 were added to the soluble chromatin and incubated with rotation at 4°C overnight. Protein G Dynabeads (Life Tech, 10004D) were washed three times with sonication buffer and then added to the soluble chromatin and antibodies, followed by incubation at 4°C for 2 h with rotation. The magnetic Dynabeads were pelleted by placing the tubes in a magnetic rack, washed, and then the bound DNA fragments were eluted. Sequencing libraries were prepared using 10 ng of purified DNA (average size 200–400 bp) using the NEBNext Ultra II Library Prep Kit for Illumina (E7645S, New England Biolabs) and subjected to 75-bp single-end sequencing on a Nova PE150 platform (Illumina).

For the analysis of the ChIP-seq data, the quality of each raw sequencing file (fastq) was verified with FastQC (https://www.bioinformatics.babraham.ac.uk/projects/fastqc/). All files were aligned to the reference human genome (hg19) and processed using bowtie2 (https://bowtie-bio.sourceforge.net/) for mapping and samtools (http://www.htslib.org/) for duplicate removal (rmdup), sorting (sort) and indexing (index). Coverage for each aligned ChIP-seq dataset (.bam) were computed with deeptools (https://deeptools.readthedocs.io/en/latest/index.html) and normalized using total read count for each sample. Coverage data were exported as bigwig (file format) for further processing. Averaged ChIP-seq profiles were generated using the R package ggplot2. The x axis represents genomic position relative to DSB and the y axis represents the mean coverage at each bp. Heatmaps were generated by computeMatrix tool from deepTools.

ChIP-qPCR was performed using primers shown in Supplementary Table S1. Data were analyzed using CFX Manager Software (Bio-Rad). The fold change of protein-binding DNA was calculated by using the following steps: (i) calculate the ΔCt values from the ChIP (Ct_1_) and input (Ct_2_) C_t_ values by using the formula ΔCt = Ct_1_ – Ct_2_; (ii) calculate the fold change from the corresponding ΔCt values by using the formula Fold change = 2^−ΔCt^; (iii) normalize the fold change in the ESCO2 KD cells with that of the WT cells and use GraphPad to generate the heat map of these normalized fold change results and (iv) perform two-way ANOVA from multiple independent biological replicates to obtain and analyze the *P* values.

### Duolink proximity ligation assay (PLA)

PLAs were performed to examine the *in situ* interaction between MDC1 and ESCO2 WT or the ESCO2-2A mutant in ESCO2 KD HCT116 cells transfected with Flag-ESCO2 WT and Flag-ESCO2-2A plasmids. The assay was performed using the Duolink^®^ In Situ PLA^®^ kit (DUO92101, Sigma-Aldrich) as described previously (28).

### Nude mice xenograft assay

Female BALB/c nude mice at 6–8 weeks of age were purchased from Beijing Vital River Laboratory Animal Technology. HCT116 ESCO2 WT or KD cells (5 × 10^6^ cells) were injected subcutaneously into both flanks, tumor size was measured every 3 days using a caliper, and tumor volume was calculated using the following formula: volume = (length × width^2^)/2. At 24 days post-injection, tumors were dissected and weighed. The nude mice tumorigenesis assay was approved by the IACUC of the Center for Experimental Animal Research (China) and Peking University Laboratory Animal Center (IACUC No. LSCZhengX-2–1) and performed in accordance with the ‘Guide for the Care and Use of Laboratory Animals’.

### Statistical analysis

All statistical results are presented as the mean ± SD or mean ± SEM, as indicated. *P* values (* *P* < 0.05, ** *P* < 0.01, *** *P* < 0.001, **** *P* < 0.0001) were obtained using Student's *t* test (two-tailed) or one/two-way ANOVA from multiple independent biological replicates.

## RESULTS

### ESCO2 is involved in DSB repair and is essential for maintaining genome stability

**Figure 1.**
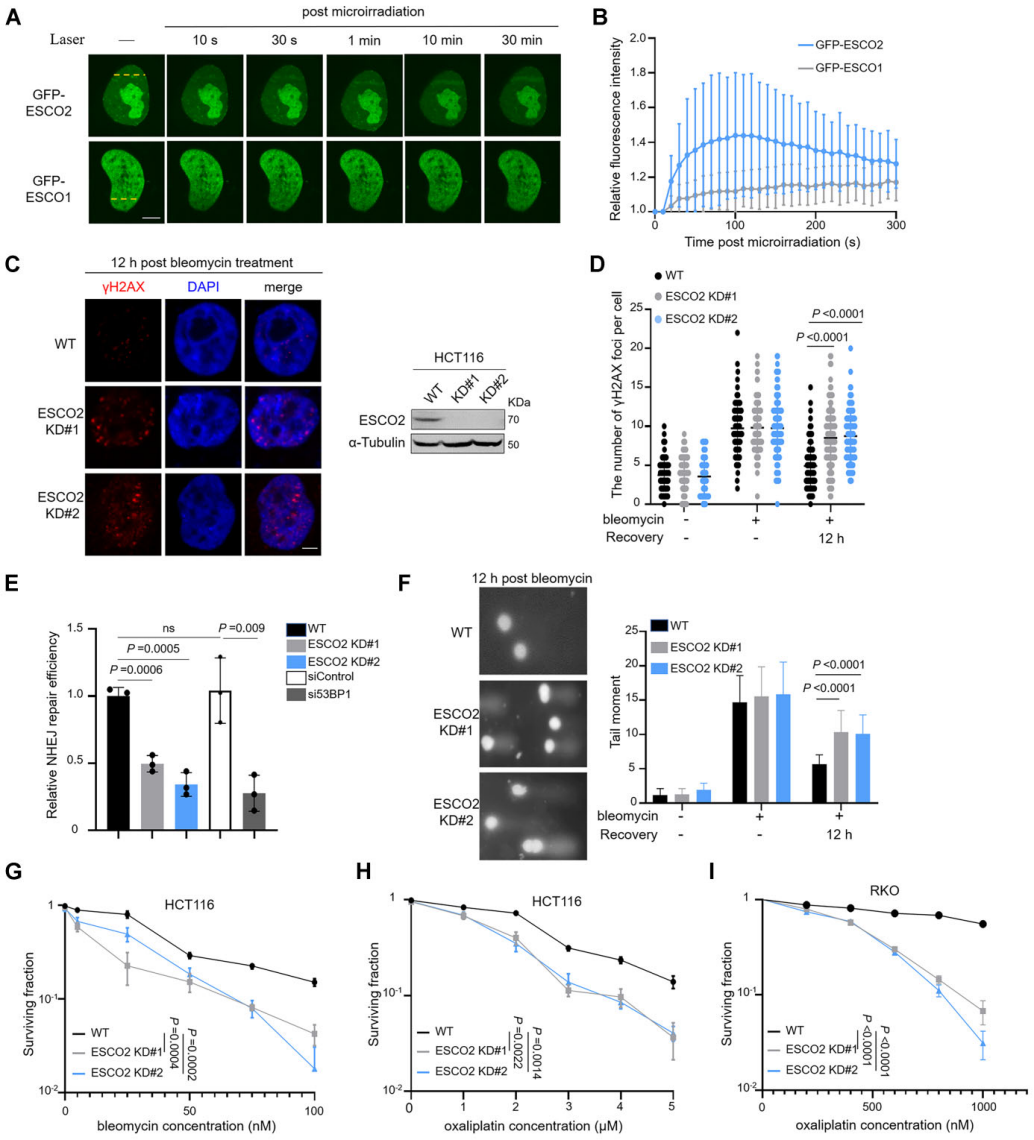
ESCO2 is involved in DSB repair and genomic stability maintenance. (A, B) HeLa cells transfected with GFP-ESCO2 or GFP-ESCO1 were subjected to a laser microirradiation assay. GFP fluorescence was detected by fluorescence microscopy at the indicated time points. Representative images are shown (**A**). Scale bar, 2 μm. Relative fluorescence intensity time plots showing that ESCO2 is recruited to damage site (**B**). The graphs show mean ± SEM; *n*= 10 for each group. (**C**, **D**) Wild-type (WT) and ESCO2 knockdown HCT116 cells treated without (–) or with (+) bleomycin (5 μM, 2 h) were subjected to immunofluorescence assays and γH2AX foci were detected. ESCO2 knockdown was confirmed by western blotting. Representative images from three independent experiments are shown. Scale bar, 2 μm. The graphs show mean ± SEM; *n*= 100 for each group. (**E**) NHEJ efficiency was determined in ESCO2 WT, ESCO2 KD, siControl-transfected and si53BP1-transfected HCT116 cells. NHEJ efficiency is presented as relative to that of WT HCT116 cells. (**F**) A neutral comet assay was performed using WT and ESCO2 knockdown HCT116 cells treated with (+) or without (–) bleomycin; the representative images are shown. The quantified tail moments are shown for each group (*n* = 50). The graph shows mean ± SEM. (**G–I**) The sensitivities of WT and ESCO2 knockdown HCT116 or RKO cells to bleomycin and oxaliplatin were examined by colony formation assay and plotted as the fraction of surviving cells relative to the number of untreated cells. Statistical analysis in (D–I) was performed using a Student's *t* test.

Cells derived from patients with *ESCO2* mutation-induced RBS are more sensitive to ionizing radiation (IR)- and mitomycin C-induced DNA damage (29). To confirm whether ESCO2 is involved in the DNA damage response (DDR), we performed a laser microirradiation assay coupled with live imaging of protein redistribution and found that GFP-ESCO2, but not GFP-ESCO1, was rapidly and robustly recruited to the micro-irradiated region (Figure 1A and Supplementary Figure S1A), which was detectable approximately 10–30 s after microirradiation (Figure 1B). These observations suggest that ESCO2 is involved in the DDR.

It has been well established that overactivation of DDR pathway proteins results in resistance to chemotherapy or radiotherapy cancer treatments, and loss of DDR elements increases sensitivity to DNA damage agents (30). Analyses of TCGA and GTEx databases showed that the expression level of ESCO2 in colorectal cancer tissues was higher than that in normal tissues (Supplementary Figure S1B). Moreover, the expression level of ESCO2 was correlated with those of 53BP1 and BRCA1, which are both essential for DSB repair (Supplementary Figure S1C). This analysis from cancer samples implies that ESCO2 is associated with DSB repair in colorectal cancers. Therefore, we tested ESCO2 expression levels in three colorectal cancer cell lines (HCT116, LOVO and RKO) and two normal colorectal epithelial cell lines (NCM460 and FHC). As expected, the abundance of ESCO2 was higher in cancer cells relative to normal cells (Supplementary Figure S1D). To further investigate the role of ESCO2 in the DDR in colorectal cancer, we stably knocked down ESCO2 in HCT116 cells (ESCO2 KD HCT116) and monitored γH2AX foci, a biomarker of DSB damage, using an immunofluorescence assay 12 h after bleomycin treatment. Relative to wild-type (WT) cells, cells with depleted ESCO2 showed an increased number of γH2AX foci after a 12 h recovery (Figure 1C, D); furthermore, we achieved similar results in RKO cells (Supplementary Figure S2A). Next, we examined the effect of ESCO2 on the DSB repair efficiency. In addition to showing that overexpression of ESCO2 promoted HR efficiency (Supplementary Figure S2C), which is consistent with a previous study (16), we also found that NHEJ repair efficiency decreased in ESCO2-depleted HCT116 cells but increased with ESCO2 overexpression using 53BP1 KD cells as a negative control (Figure 1E and Supplementary Figure S2B, C). Additionally, we performed a neutral comet assay using WT and ESCO2 KD HCT116 cells treated with or without bleomycin. The results showed that ESCO2 deficiency increased the length of comet tails after a 12 h recovery (Figure 1F). These results indicate that ESCO2 is involved in DSB repair and genome stability maintenance. Oxaliplatin, which can induce DNA damage, is a third-generation platinum drug used as a first-line chemotherapy in colorectal cancer. We examined the effect of ESCO2 depletion on cell survival following bleomycin or oxaliplatin treatment in both HCT116 and RKO cells. Relative to WT HCT116 and/or WT RKO cells, knockdown of ESCO2 rendered both HCT116 and RKO cells more sensitive to bleomycin and oxaliplatin (Figure 1G–I). Collectively, these results suggest that ESCO2 promotes chemotherapeutic drug-induced DDR in colorectal cancer cells.

### ATM regulates the recruitment of ESCO2 at DSB sites by phosphorylating ESCO2 S196 and T233 residues

**Figure 2.**
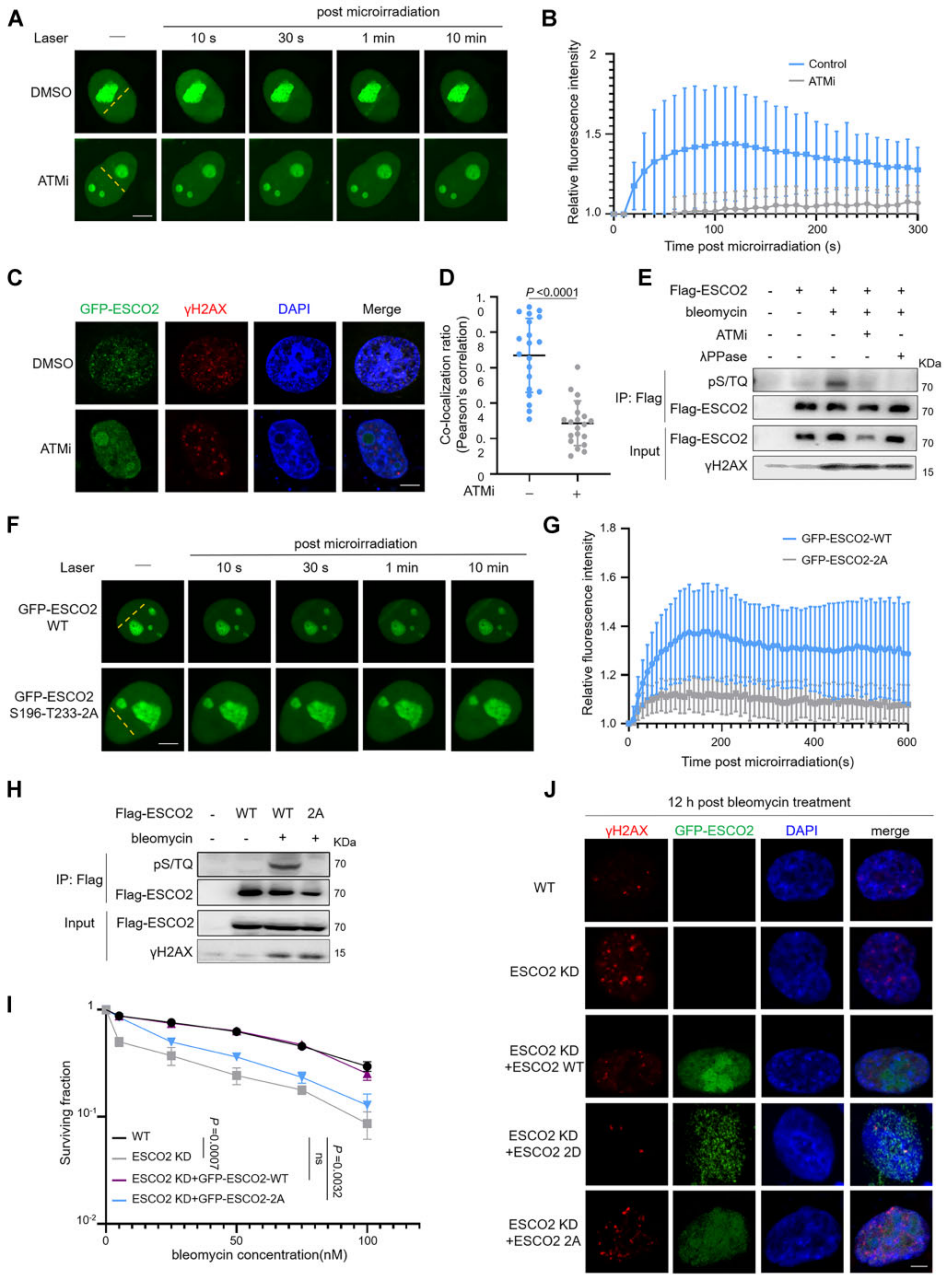
ATM regulates the recruitment of ESCO2 to DSB sites by phosphorylating S196 and T233 residues. (**A**, **B**) HeLa cells were transfected with GFP-ESCO2 for 36 h, followed by treatment with 10 μM ATM inhibitor KU55933 for 2 h, after which laser microirradiation assays were performed. Scale bar, 2 μm. The graphs show mean ± SEM; *n* = 10 for each group. (**C**, **D**) HeLa cells transfected with GFP-ESCO2 were treated with ATM inhibitor KU55933 for 2 h followed by bleomycin treatment (5 μM, 2 h), and then immunofluorescence assays were performed to examine the co-localization of ESCO2 and γH2AX foci. Representative images are shown. Scale bar, 2 μm. The co-localization ratio (Pearson's correlation) was analyzed using ImageJ. The graphs show mean ± SEM; *n* = 20 for each group. Statistical analysis was performed using a Student's *t* test. (**E**) HEK293T cells were transfected with the indicated plasmids. At 36 h after transfection, cells were treated with (+) or without (–) 10 μM ATM inhibitor (ATMi) for 2 h. The cells were then treated with (+) or without (–) bleomycin and lysed. Next, immunoprecipitation analysis was performed using anti-Flag M2-agarose beads, eluted using Flag peptide, and then incubated with (+) or without (–) λ-PPase at 37°C for 0.5 h. (**F**, **G**) HeLa cells transfected with GFP-ESCO2 wild-type (WT) or GFP-ESCO2 S196-T233-2A plasmid were subjected to a laser microirradiation assay. GFP fluorescence was detected by fluorescence microscopy at the indicated time points. Representative images are shown. Scale bar, 2 μm. The graph shows mean ± SEM; *n* = 10 for each group. (**H**) HEK293T cells were transfected with the indicated plasmids for 36 h and then treated with (+) or without (–) bleomycin, and the effect of DNA damage on WT or mutant ESCO2 phosphorylation was assessed by IP analysis using the indicated antibodies. (**I**) WT RKO cells, ESCO2 knockdown RKO cells, and ESCO2 knockdown RKO cells transfected with the indicated plasmids were treated with bleomycin. The sensitivities of cells are plotted as the fraction of surviving cells relative to the number of untreated cells. Statistical analysis was performed using a Student's *t* test. (**J**) HCT116 cells were transfected with the indicated plasmids. At 36 h after transfection, the cells were treated with bleomycin for 2 h followed by culture in fresh medium for 12 h. Immunofluorescence assays were performed to examine the γH2AX foci. Scale bar, 2 μm.

To determine the mechanism by which ESCO2 is recruited to DNA damage sites, we assessed whether ESCO2 recruitment is dependent on the upstream kinases, such as ATM, ATR or DNA-PKcs. Cells transfected with GFP-ESCO2 were treated with specific inhibitors targeting ATM (ATMi, KU55933), ATR (ATRi, VE821) or DNA-PKcs (DNA-PKcsi, NU7441), and the recruitment ability of GFP-ESCO2 to the laser tracks after microirradiation was examined. Interestingly, we found that ATM inhibition reduced the recruitment of ESCO2 (Figure 2A, B and Supplementary S3A); however, neither ATR nor DNA-PKcs inhibition affected ESCO2 recruitment (Supplementary Figure S3B). Consistent with this observation, the co-localization ratio of ESCO2 and γH2AX foci after bleomycin treatment was reduced in cells treated with ATMi (Figure 2C, D). Previous studies revealed the preference of PIKKs family members (including ATM, ATR and DNA-PKcs) for phosphorylating a serine or threonine followed by a glutamine (S/TQ; (5)). Here, using a phospho-specific S/TQ antibody that specifically recognizes proteins phosphorylated on S/TQ motifs, we observed that the phosphorylation of ESCO2 responding to DNA damage significantly decreased when ATM was inhibited. Cells treated with λ phosphatase that can dephosphorylate the phosphorylated serine, threonine and tyrosine residues were used as negative controls (Figure 2E).

To identify the phosphorylation sites of ESCO2 by ATM, we generated a series of mutation constructs by mutating S/TQ motif residues S50, S196 and T233 to alanine, which served to mimic the non-phosphorylation status of these residues. We then tested whether these mutants could be recruited to DSB sites. Among the mutants examined, compared with GFP-ESCO2 WT, the double mutant GFP-ESCO2-S196-T233-2A (designated as 2A in figures) showed a clear reduction in its recruitment to the laser track (Figure 2F, G and Supplementary Figure S4A, B) and had no impact on the acetyltransferase activity of ESCO2 (Supplementary Figure S4C). Moreover, the phosphorylation level of the GFP-ESCO2-S196-T233-2A mutant was no longer detected after bleomycin treatment (Figure 2H). The cell survival assay results showed that overexpression of GFP-ESCO2 WT, but not GFP-ESCO2-S196-T233-2A, reversed bleomycin-hypersensitivity in ESCO2-depleted cells (Figure 2I). We also found a reduced number of γH2AX foci in both the ESCO2-depleted cells overexpressing GFP-ESCO2 WT and in the GFP-ESCO2-S196-T233-2D mutant, which mimicked the continuous phosphorylation state of ESCO2, but not in the GFP-ESCO2-S196-T233-2A mutant (Figure 2J). These results indicate that ATM-mediated phosphorylation of ESCO2 on its S196 and T233 residues is essential for its recruitment to DSB sites.

### MDC1 interacts with and mediates the recruitment of ESCO2 to DSB sites

**Figure 3.**
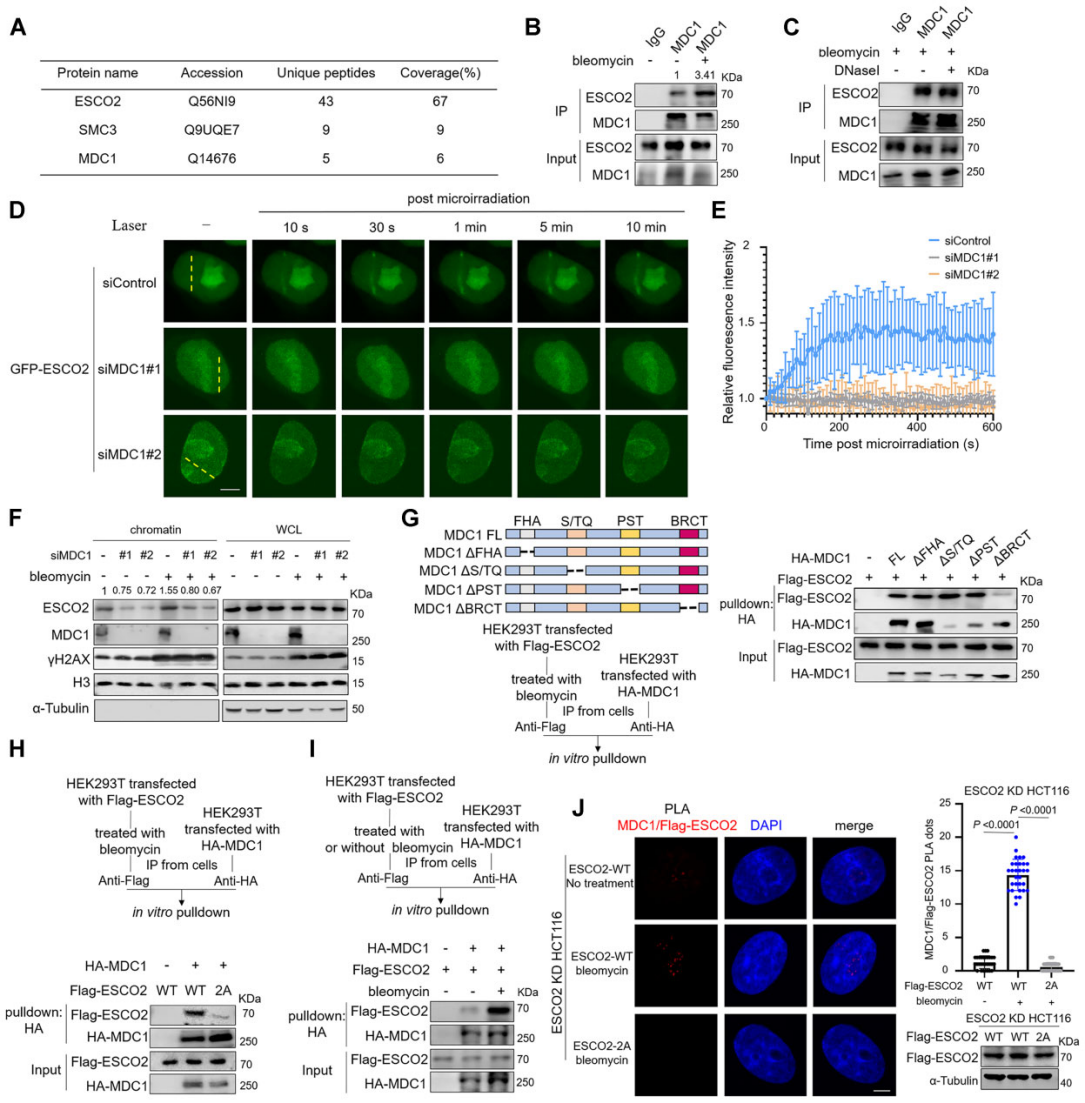
MDC1 interacts with ESCO2 and mediates the recruitment of ESCO2 to DSB sites. (**A**) Tandem affinity purification was performed using HEK293T cells transfected with Flag-ESCO2. At 36 h after transfection, the cells were treated with bleomycin, and Flag-ESCO2 was immunopurified with Flag M2-agarose beads and eluted using Flag peptide. The eluate was analyzed using mass spectrometric analysis. Major hits from the mass spectrometric analysis are shown in the table. (**B**) The effect of bleomycin-induced DNA damage on the MDC1-ESCO2 interaction was assessed by co-IP in HCT116 cells with (+) or without (–) bleomycin. (**C**) The IP assay was performed with bleomycin-treated HCT116 cells to eliminate the interference of damaged DNA. The cell lysate was treated for 30 min at 4°C with (+) or without (–) DNaseI, which nonspecifically cleaves DNA to oligonucleotides. (D, E) HCT116 cells co-transfected with GFP-ESCO2 and siControl/siMDC1 were subjected to a laser microirradiation assay. GFP fluorescence was detected by fluorescence microscopy at the indicated time points. Representative images are shown (**D**). Scale bar, 2 μm. Relative fluorescence intensity time plots showing that ESCO2 is recruited to damage sites (**E**). The graphs show mean ± SEM; *n* = 10 for each group. (**F**) Control and MDC1 KD HCT116 cells were treated with (+) or without (–) bleomycin, after which the chromatin was isolated and analyzed using the indicated antibodies. (**G**) HEK293T cells were transfected with the indicated plasmids for 36 h followed by *in vitro* pulldown. The cells used for purifying Flag-ESCO2 were treated with bleomycin. The Flag-ESCO2 protein was immunoprecipitated with Flag-M2 beads and eluted using Flag peptide. The HA-MDC1 protein was immunoprecipitated with anti-HA antibody and Protein G beads. Next, the beads with HA-MDC1 protein were incubated with the eluted ESCO2 protein at 4°C for 2 h. (**H**) HEK293T cells were transfected with HA-MDC1, Flag-ESCO2 WT, and S196-T233-2A followed by *in vitro* pulldown to examine the interaction between MDC1 and ESCO2 WT or mutant. The phosphorylated ESCO2 proteins were enriched from HEK293T cells treated with bleomycin. (**I**) HEK293T cells transfected with HA-MDC1 and Flag-ESCO2 WT were subjected to an *in vitro* pulldown assay to examine the effect of ESCO2 phosphorylation on its interaction with MDC1. The phosphorylated and non-phosphorylated ESCO2 proteins were immunoprecipitated from HEK293T cells treated with (+) or without (–) bleomycin, respectively. (**J**) The effect of bleomycin treatment and phosphorylation of ESCO2 on the interaction between MDC1 and Flag-ESCO2 was detected in HCT116 KD cells transfected with Flag-ESCO2 WT or Flag-ESCO2-2A by PLAs. Representative images are shown, and 30 cells were counted in each group to analyze the number of PLA dots. The graphs show mean ± SEM; statistical analysis was performed using a Student's *t* test. The expression levels of Flag-ESCO2 and Flag-ESCO2-2A in PLA was detected by immunoblotting. Cells in (A–C) and (G–I) were treated with 5 μM bleomycin for 2 h.

To further elucidate the mechanism underlying the recruitment of ESCO2 to DNA damage sites, we performed an immunoprecipitation (IP) assay followed by mass spectrometry to identify ESCO2-interacting proteins in response to DNA damage. MDC1, which is a scaffold protein involved in the early steps of the DDR, was shown to be a potential partner of ESCO2 (Figure 3A and Supplementary Table S2); this interaction between ESCO2 and MDC1 was further confirmed by co-IP assay, which revealed an enhanced interaction in bleomycin-treated cells (Figure 3B and Supplementary Figure S5A) that was not mediated by DNA (Figure 3C). To examine whether ESCO2 regulated DSB repair through its association with MDC1, we performed laser microirradiation assays using HCT116 cells transfected with siControl or siMDC1. The results showed that ESCO2 was no longer recruited to damage sites in MDC1-depleted cells (Figure 3D, E and Supplementary Figure S5B). This phenomenon was confirmed by chromatin isolation assay, in which the fold change of ESCO2 level on chromatin in response to bleomycin treatment is 1.55 in siControl cells (lane 4 versus lane 1) and are 1.07 (lane 5 versus lane 2) and 0.94 (lane 6 versus lane 3) respectively in the two MDC1-depleted cell lines (Figure 3F). These results indicate that ESCO2 is recruited to DSB sites in an MDC1-dependent manner.

MDC1 is composed of several distinct domains and regions that can recognize and interact with its partners recruited to DSB sites. We next mapped the critical domain of MDC1 interacting with ESCO2 using an *in vitro* pulldown assay (Figure 3G) and discovered that, surprisingly, the BRCT domain of MDC1 is responsible for its interaction with ESCO2. Since the BRCT domain acts as a phosphopeptide-binding domain, we needed to clarify whether ESCO2 phosphorylation is critical for its binding to MDC1; therefore, we tested the direct interaction between MDC1 and ESCO2 WT or the ESCO2-S196-T233-2A mutant. As expected, relative to ESCO2 WT, the interaction with MDC1 was largely reduced in the ESCO2-S196-T233-2A mutant (Figure 3H). We also found that bleomycin-induced DNA damage promoted the association between ESCO2 and MDC1 (Figure 3I). Furthermore, the PLA results showed that the *in situ* interaction between MDC1 and ESCO2 was enhanced in response to bleomycin treatment and decreased when the phosphorylation sites were mutated (Figure 3J). Collectively, these results indicate that the BRCT domain of MDC1 recognizes phosphorylated ESCO2 after DNA damage and recruits ESCO2 to the damage sites.

### ESCO2 promotes the formation of 53BP1 foci

**Figure 4.**
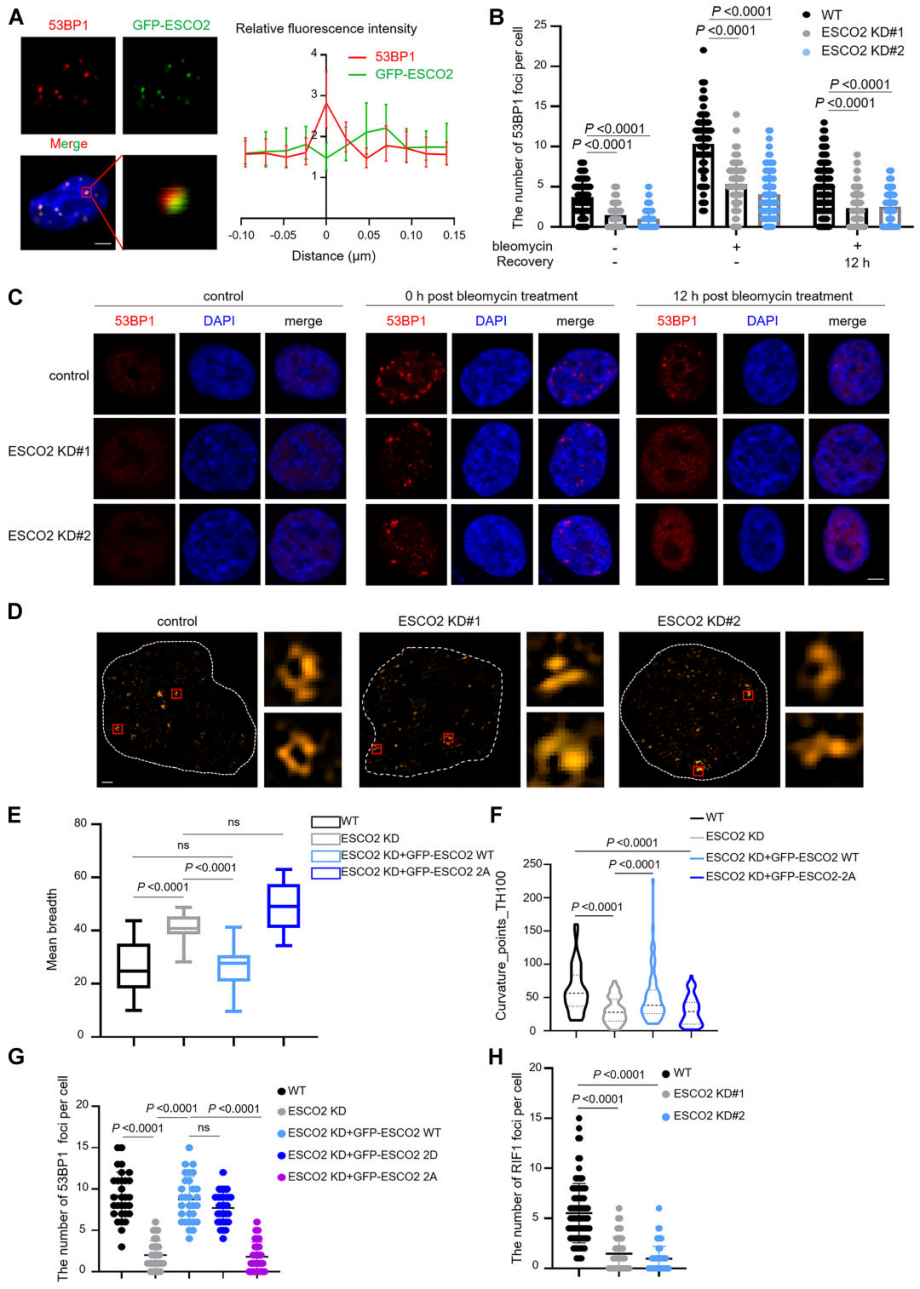
ESCO2 promotes the formation of 53BP1 foci and NHEJ efficiency. (**A**) Colocalization of ESCO2 and 53BP1 in HCT116 cells after bleomycin treatment was monitored by confocal microscopy. Representative images are shown. Scale bar, 2 μm. Quantification of co-localized foci is shown as mean ± SD from *n* = 21 cells. (**B**, **C**) Wild-type (WT) and ESCO2 knockdown HCT116 cells treated with (+) or without (–) bleomycin (5 μM, 2 h) were subjected to immunofluorescence assays at the indicated time points after DNA damage, and the effect of ESCO2 depletion on 53BP1 foci formation was examined. Representative images from three independent experiments are shown. Scale bar, 2 μm. The graphs show mean ± SEM; *n* = 100 for each group. (**D**) 3D-SIM of 53BP1 foci in WT and ESCO2 knockdown HCT116 cells treated with bleomycin (5 μM, 2 h). Scale bar, 1 μm. (E, F) QUANTEX analysis of mean-breadth (**E**) and Curvature_points_TH100 (**F**) of 53BP1 in WT, ESCO2 knockdown HCT116 cells, and ESCO2 knockdown HCT116 cells transfected with GFP-ESCO2 WT or GFP-ESCO2 S196-T233-2A plasmid. The graphs show mean ± SEM; *n*= 50 for each group. (**G**) HCT116 cells transfected with the indicated plasmid were treated with bleomycin for 2 h. Immunofluorescence assays were then performed to examine the 53BP1 foci. Scale bar, 2 μm. The graph shows mean ± SEM; *n*= 30 for each group. (**H**) WT and ESCO2 knockdown HCT116 cells were treated with bleomycin for 2 h, and then the RIF1 foci were examined by immunofluorescence assays. Scale bar, 2 μm. The graph shows mean ± SEM; *n*= 90 for each group. Statistical analysis in panels (B), (E), (F), (G) and (H) was performed using a Student's *t* test.

Since a previous study revealed the role of ESCO2 in HR repair (16), we then primarily investigated the mechanism by which ESCO2 promoted NHEJ repair and maintained genome stability. The immunofluorescence assay in cells treated with bleomycin showed that ESCO2 formed damage-induced foci (Supplementary Figure S6A); additionally, the localization of ESCO2 at DSBs was devoid of 53BP1 foci, and analysis of protein intensities revealed that ESCO2 and 53BP1 displayed mutually exclusive but adjacent localization patterns (Figure 4A). According to this observation, we speculated that ESCO2 might regulate the formation of 53BP1 foci; therefore, we measured the number of 53BP1 foci in ESCO2 KD HCT116 cells. Surprisingly, relative to WT cells, the number of 53BP1 foci decreased significantly in ESCO2-depleted cells—regardless of bleomycin treatment—with similar results in RKO cells (Figure 4B, C and Supplementary Figure S6B). Furthermore, depletion of ESCO2 did not reduce the number of RNF8, RNF168, H4K20me2/3, and FK2 foci (Supplementary Figure S6C). These results suggest that ESCO2 is important for the formation of 53BP1 foci.

A previous study has shown that 53BP1 foci consist of four to seven nano-domains (53BP1-NDs), which assemble to form microdomains (53BP1-MDs) (13). We used 3D-SIM super-resolution microscopy to observe the effect of ESCO2 on the formation of 53BP1-MDs and found that ESCO2 deficiency disrupted the formation of 53BP1-MDs into disordered shapes (Figure 4D–F). QUANTEX three-dimensional structure analysis of 53BP1-MDs derived from ESCO2 KD HCT116 and RKO cells revealed an increase in mean breadth and a reduction in Curvature_point_TH100 (Figure 4E, F and Supplementary Figure S6D, E), which indicated a collapse of the circularization of 53BP1-MDs. In ESCO2 KD cells, reintroduction of WT ESCO2, but not the ESCO2-S196-T233-2A mutant that could not be recruited to damage sites, could reshape the high-order organization of 53BP1-MDs (Figure 4E, F). Moreover, Imaris image analysis was used to reconstruct the super-resolution fluorescence images of 53BP1 and GFP-ESCO2 and calculate the distance of either GFP-ESCO2 WT or 2A to the 53BP1-MDs. This analysis revealed that, in WT cells without ATM inhibitor treatment, GFP-ESCO2 was located either inside or at the periphery of 53BP1-MDs, but in cells treated with ATM inhibitor, it was far away from 53BP1-MDs. Additionally, GFP-ESCO2 2A showed the same pattern as that of GFP-ESCO2 in ATM inhibitor-treated cells (Supplementary Figure S6F). These results explain the exclusive localization patterns seen in confocal imaging (Figure 4A) and why ESCO2 WT, but not the ESCO2-S196-T233-2A mutant, could remold the high-order organization of 53BP1-MDs (Figure 4E, F). In line with these observations, 53BP1 could not form repair foci in ESCO2-depleted cells transfected with the ESCO2-S196-T233-2A mutant (Figures 4G and Supplementary Figure S6G). Because 53BP1 recruits RIF1 to damage sites to prevent resection and channels DSB repair to the NHEJ pathway (9,11), we examined the number of RIF1 foci and found that the recruitment of RIF1 was restrained in ESCO2 knockdown cells (Figures 4H and Supplementary Figure S6H). These results indicate that ESCO2 regulates the formation of 53BP1 foci and promotes NHEJ repair.

### ESCO2 regulates the chromatin structure around DSB sites by acetylating SMC3 K105/106

**Figure 5.**
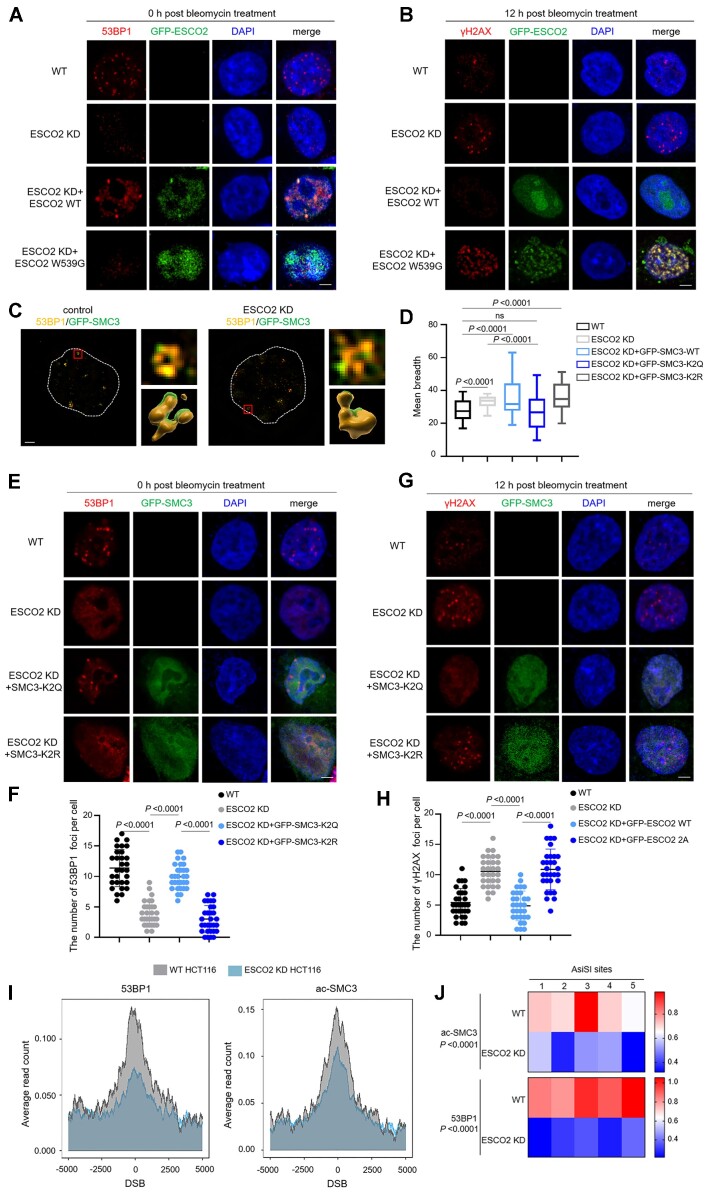
ESCO2 regulates the chromatin structure around DSB sites by acetylating SMC3 K105/106. (A, B) ESCO2 WT, ESCO2 knockdown HCT116 cells, and ESCO2 knockdown HCT116 cells transfected with GFP-ESCO2 WT or GFP-ESCO2-W539G were treated with bleomycin for 2 h. To examine the 53BP1 foci, immunofluorescence assays were performed immediately after bleomycin treatment (**A**). Scale bar, 2 μm. To examine the γH2AX foci, the cells were cultured in fresh medium for 12 h after bleomycin treatment (5 μM, 2 h) and immunofluorescence assays were then performed (**B**). Scale bar, 2 μm. (**C**) 3D-SIM of 53BP1 and GFP-SMC3 foci in wild-type (WT) and ESCO2 knockdown HCT116 cells treated with bleomycin. Scale bar, 1 μm. 3D reconstruction using Imaris. (**D**) QUANTEX analysis of mean-breadth of 53BP1 in WT HCT116 cells, ESCO2 knockdown HCT116 cells, and ESCO2 knockdown HCT116 cells transfected with GFP-SMC3-WT, GFP-SMC3-K2Q or GFP-SMC3-K2R plasmid. (**E**, **F**) ESCO2 knockdown HCT116 cells transfected with the indicated plasmids and WT HCT116 cells were treated with bleomycin for 2 h. Immunofluorescence assays were performed to examine the 53BP1 foci. Representative images from three independent experiments are shown. Scale bar, 2 μm. The graphs show mean ± SEM; *n* = 30 for each group. (**G**, **H**) ESCO2 knockdown HCT116 cells transfected with the indicated plasmids were treated with bleomycin for 2 h, and the cells were then cultured in fresh medium for 12 h. Immunofluorescence assays were then performed to examine the γH2AX foci. Representative images from three independent experiments are shown. Scale bar, 2 μm. The graphs show mean ± SEM; *n* = 30 for each group. (**I**) Average profile for 53BP1 and ac-SMC3 in ESCO2 WT and ESCO2-depleted HCT116 cells. ChIP-seq analyses of WT and ESCO2-depleted HCT116 cells after 4-OHT treatment (500 nM, 4 h), using anti-53BP1 and anti-ac-SMC3 antibodies. Averaged 53BP1 and ac-SMC3 signals over a 10-kb region flanking annotated AsiSI sites are shown. (**J**) ChIP analysis was performed in AsiSI–ER-HCT116 cells after 4 h 4-OHT treatment, using anti-ac-SMC3 and anti-53BP1 antibodies as indicated. ac-SMC3 and 53BP1 enrichment was assessed by qPCR amplification using proximal primers of AsiSI sites. Statistical analysis was performed using two-way ANOVA. Statistical analysis in panels (D), (F) and (H) was performed using a Student's *t* test.

The acetylation of SMC3 by ESCO2 is related to the establishment of cohesion and the increased stability of the cohesin-chromatin association (23,24,31). Furthermore, the cohesin-mediated DNA loop extrusion is involved in DSB repair (32). Our IP-MS assay that identified the ESCO2-SMC3 interaction (Figure 3A) led us to speculate that ESCO2 promoted DSB repair by acetylating SMC3 and stabilizing cohesin structure. An ESCO2 W539G mutation has been identified in RBS patients, which results in a loss of acetyltransferase activity (33). Consistent with this study, the immunofluorescence results also showed that the W539G mutant could not rescue the formation of 53BP1 foci and DSB repair capability in ESCO2-depleted cells (Figure 5A, B). Based on the above experimental results, we speculated that the ESCO2-mediated stabilization of the cohesin complex was involved in 53BP1 foci formation. To test this hypothesis, we used 3D reconstruction to monitor the high-order organization of 53BP1 and SMC3 at DSB sites. The results revealed that in WT cells, SMC3 localized at DSB breaks partially occupied by 53BP1, where they formed similar circular three dimensional structures; conversely, ESCO2 knockdown disrupted the three-dimensional organization of SMC3 and 53BP1 (Figure 5C, D). We mutated SMC3 K105/106 to glutamine (K2Q), which mimicked the hyper-acetylated state of SMC3, or to arginine (K2R), which mimicked the unacetylated state; we then analyzed the mean breadth of the 53BP1-MDs. Only the SMC3-K2Q mutant rescued the three-dimensional organization of the 53BP1-MDs (Figure 5D), and an increased number of 53BP1 foci was detected in ESCO2-depleted cells re-expressed with SMC3-K2Q (Figure 5E, F). In keeping with this data, we observed a decrease in the number of γH2AX foci after 12 h recovery in ESCO2 knockdown cells re-expressed with SMC3-K2Q (Figure 5G, H), suggesting that ESCO2-catalyzed SMC3 acetylation promoted the repair efficiency of bleomycin-induced DSBs.

To investigate the recruitment of 53BP1 and cohesin around DSBs, we developed stable WT and ESCO2-depleted HCT116 cell lines expressing AsiSI, which is a restriction enzyme that targets an 8-bp recognition sequence, fused with estrogen receptor (ER) and HA tag (HA-ER-AsiSI cells). Since treatment with 4-hydroxytamoxifen (4-OHT) triggers the nuclear translocation of HA-ER-AsiSI and generates DSBs (34), we performed chromatin immunoprecipitation with sequencing (ChIP-seq) in 4-OHT-treated ESCO2 WT and ESCO2-depleted HCT116 cells using antibodies against γH2AX, 53BP1, and ac-SMC3 (acetylated SMC3 K105/106). ChIP-seq results showed that depletion of ESCO2 resulted in reduced recruitment of 53BP1 and ac-SMC3 to the DSBs and an increased level of γH2AX (Figure 5I and Supplementary Figure S7A, B). ChIP-qPCR analysis of pulled down DNA using primers proximal to a set of six AsiSI sites also showed that ESCO2 deficiency led to reductions of γH2AX, 53BP1, and ac-SMC3 around these damage sites (Figure 5J and Supplementary Table S3). Consistently, SMC3 was not recruited to DSB sites in ESCO2 deficient cells (Supplementary Figure S7C), indicating that ESCO2 stabilizes cohesin around DSB sites by acetylating SMC3. In summary, these results indicate that ESCO2 shapes the high-order chromatin structure at DSB breaks by acetylating SMC3 K105/106, which is indispensable to the assembly of 53BP1.

### Deficiency of ESCO2 leads to chemotherapy sensitivity in colorectal cancer

**Figure 6.**
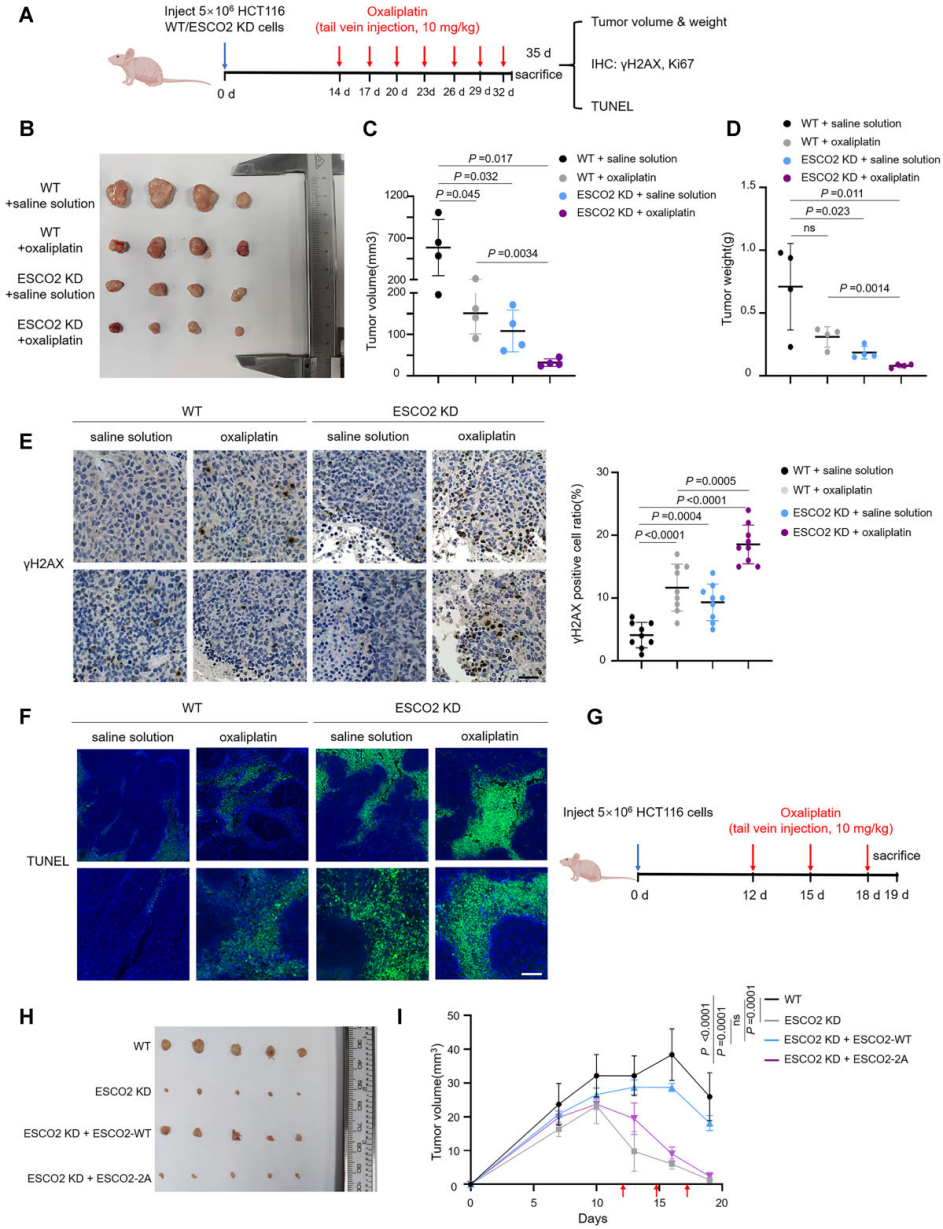
ESCO2 deficiency leads to chemotherapy sensitivity in colorectal cancer. (**A**) Schematic diagram of the human colorectal cancer nude mouse xenograft model. From day 14 to day 35 after inoculation, oxaliplatin was administered intravenously at a dose of 10 mg/kg every 3 days. (**B**) Tumors from mice of each experimental group were excised and photographed. (**C**) Colorectal tumor xenograft volumes were recorded after excision. The graph shows mean ± SEM; *n* = 4 for each group. (**D**) Tumors from each group were removed and weighed. The graph shows mean ± SEM. (**E**) The levels of γH2AX in the xenograft tumors were immunohistochemically evaluated. Scale bar, 20 μm. The graphs show mean ± SEM. (**F**) TUNEL staining of the xenograft tumors. Scale bar, 20 μm. (**G**) Schematic diagram of the human colorectal cancer nude mouse xenograft model. From day 12 to day 18 after inoculation, oxaliplatin was administered intravenously at a dose of 10 mg/kg every 3 days. The red arrow indicates the time point at which the dose was administered. *n* = 5 for each group. (**H**) Tumors from mice of each experimental group were excised and photographed. (**I**) Colorectal tumor xenograft volumes from (G) were recorded every 3 days. The graph shows mean ± SEM; *n* = 5 for each group. Statistical analysis in panels (C), (D), (E) and (I) was performed using a Student's *t* test.

To further elucidate the effect of ESCO2 on genome stability *in vivo*, we performed a xenograft nude mouse experiment by subcutaneously injecting WT or ESCO2 KD HCT116 cells into 6-week-old female BALB/c mice. From day 14 to day 35 after inoculation, oxaliplatin was administered intravenously at a dose of 10 mg/kg every 3 days, and the tumor volume was measured (Figure 6A). As expected, relative to the WT control group, ESCO2 depletion resulted in a significant decrease in tumor volume and weight in the absence of drug and rendered cells hypersensitive to oxaliplatin (Figure 6B–D). To determine the levels of γH2AX and Ki67—a marker of cell proliferation—in tumor specimens, tumors from mice in each group were collected and immunohistochemical assays (IHC) were performed. Consistently, ESCO2-depleted tumors treated with oxaliplatin showed that γH2AX was significantly increased and Ki67 was substantially reduced (Figure 6E and Supplementary Figure S8). Based on TUNEL staining results, cell apoptosis also increased in ESCO2-depleted tumors after oxaliplatin treatment (Figure 6F). To explore the physiological function of ESCO2 phosphorylation in DSB repair in mice, we injected HCT116 WT cells, ESCO2 KD cells, and ESCO2 KD cells stably expressing either ESCO2-WT or -2A into the armpits of 6-week-old female BALB/c nude mice. Starting at day 12, we administered oxaliplatin at a dose of 10 mg/kg every 3 days (Figure 6G) and measured the tumor volumes. The results showed that re-introduction of ESCO2-WT, but not the ESCO2-2A mutant, in ESCO2 KD cells reduced the chemosensitivity to oxaliplatin. Additionally, larger tumors were observed in the ESCO2-WT-expressing group relative to the ESCO2 KD and ESCO2-2A groups (Figure 6H, I). Taken together, these results suggest that ESCO2 increases the resistance of colorectal cancer cells to oxaliplatin by promoting DSB repair efficiency.

## DISCUSSION

**Figure 7.**
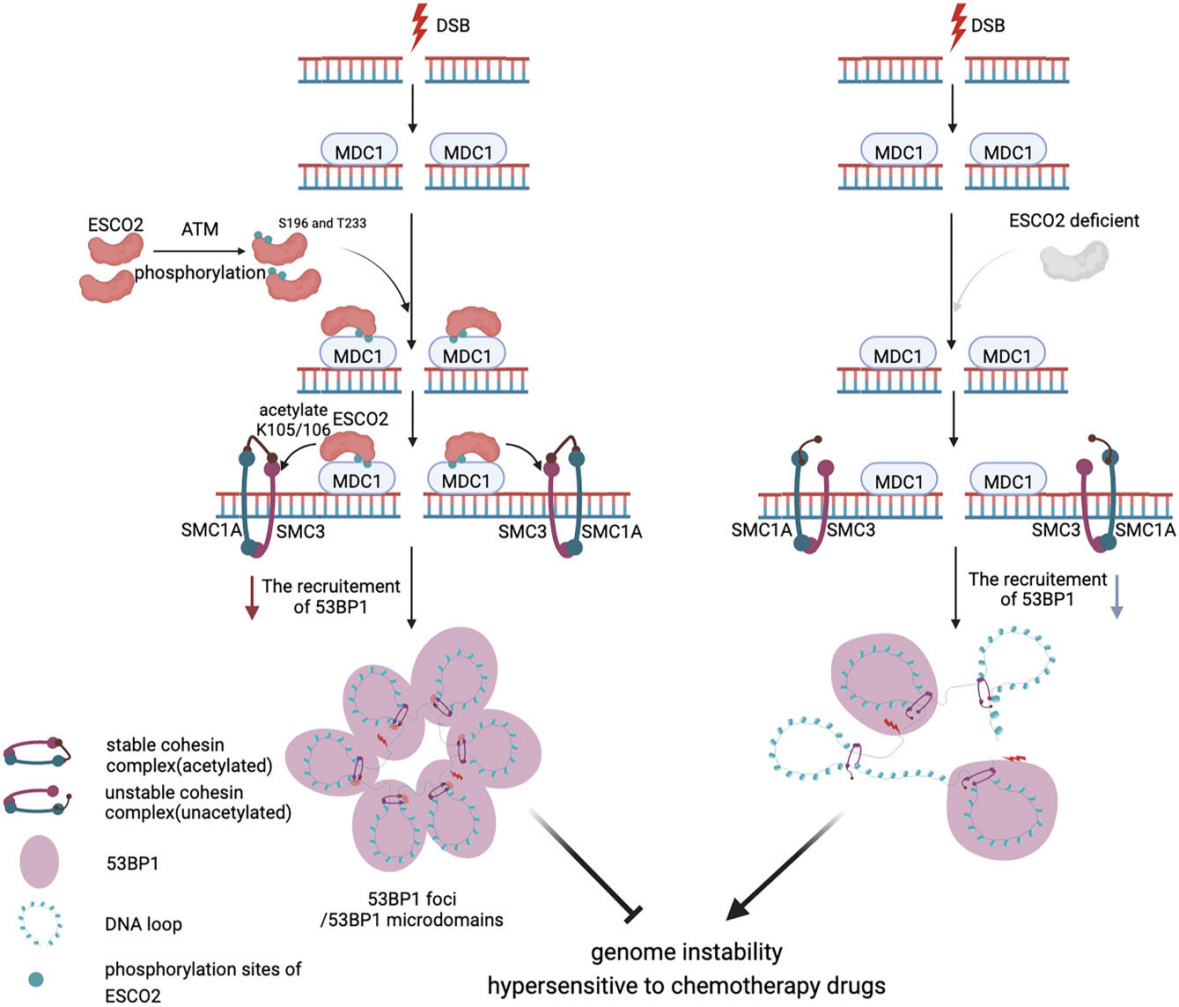
Model for the role of the ATM–ESCO2–SMC3 axis in the DNA damage response. When cells suffer from DSBs, ESCO2 S196 and T233 residues are phosphorylated by ATM. The phosphorylated ESCO2 is then recognized by MDC1 and recruited to DSB sites. ESCO2-mediated acetylation of SMC3 (a major subunit of cohesin) regulates the chromatin structure around DSB breaks and facilitates the formation of 53BP1 microdomains, which is important for NHEJ repair and genome stability. ESCO2-depleted colorectal cancer cells are more sensitive to chemotherapeutic drugs. The model was created with BioRender.com.

Our study demonstrates that ESCO2 facilitates the recruitment of 53BP1 to DSB sites and promotes the efficiency of 53BP1-directed NHEJ repair. In response to DNA damage, ESCO2 is phosphorylated by ATM kinase and recognized by MDC1, which recruits ESCO2 to DSB sites. ESCO2 acetylates SMC3 and mediates stabilization of the cohesin complex, which is essential for genome stability. Depletion of ESCO2 renders colorectal cancer cells hypersensitive to chemotherapeutic drugs (Figure 7).

A range of experimental evidence supports the model that the cohesin complex and CCCTC-binding factor (CTCF) mediate DNA loop extrusion, which organizes the chromosome (35). The three-dimensional structure of the eukaryotic genome impacts and is regulated by DNA metabolism pathways such as the DNA damage response (36); for instance, chromatin at DSB sites compacts in a unique manner that is distinguishable from undamaged chromatin in living cells (37). Piazza *et al.* showed that cohesin promotes HR repair by regulating the HR pathway (16). Consistently, we also found that overexpression of ESCO2 promoted HR repair and BRCA1 could not form damage-induced foci in ESCO2 KD cells (Supplementary Figure S2C, D). This finding suggests that the ESCO2-stabilized cohesin complex plays an important role in the HR repair pathway. Strikingly, our results showed that ESCO2 played an indispensable role in NHEJ repair, since ESCO2 overexpression increased NHEJ efficiency while ESCO2 depletion decreased NHEJ efficiency (Supplementary Figure S2C, Figure 1E). Previous work showed that 53BP1 forms microdomains that stabilize chromatin topology at DSB sites (13). DSBs induce a genome-wide increase in cohesin to isolate damaged regions from adjacent chromatin (32). Consistently, we also found that SMC3, a major subunit of cohesin, could be recruited to the DSB site (Figure 5I and Supplementary Figure S7C). Furthermore, we identified ESCO2 as a regulator for cohesin-dependent DSB-induced chromatin structure dynamics, which is important for the recruitment of cohesin and 53BP1 to DSB sites and the formation of 53BP1-MDs. Moreover, our results suggest that DNA damage-stimulated ESCO2 phosphorylation at residues S196 and T233 by ATM is essential for the recruitment of ESCO2 to DSB sites, which is consistent with the model indicating that ATM activity is required for loop extrusion at DSBs (32). Additionally, we showed that phosphorylated ESCO2 is recognized by the phosphopeptide-binding BRCT domain of MDC1 and recruited to the damage sites in an MDC1-dependent manner. Furthermore, a previous study revealed that the BRCT domain of MDC1 is critical for its binding to γH2AX (38). These data suggest that the BRCT domain of MDC1 proteins serves as a key functional domain to recognize different proteins; however, the detailed mechanism underlying the simultaneous binding of phosphorylated ESCO2 and γH2AX to the BRCT domain of MDC1 remains to be elucidated by structural biology. Taken together, our study added an additional layer of DSB-induced chromatin dynamics through the ATM–ESCO2–SMC3 axis. Collectively, our results combined with previous studies indicate that damage-induced changes in chromatin structure are essential for the cellular response to DNA damage and the recruitment of repair factors like 53BP1, and in turn the recruitment of repair proteins at DSBs regulates the chromatin structure around break sites and safeguards genome integrity.

Previous research has revealed that cancer cells produce elevated levels of ROS (39), which has been reported to directly induce oxidative DNA damage, and the failure of base excision repair leads to the generation of DSBs (40,41). Moreover, dysfunction of Eco1 and cohesin in *Saccharomyces cerevisiae* leads to ROS overproduction (42). Here, we showed that depletion of ESCO2 reduced tumor growth and increased the γH2AX level in the absence of chemotherapy reagents, suggesting that the endogenous ROS-induced DSB damage cannot be properly repaired in ESCO2-depleted cells. Moreover, ESCO2-depleted cells have been reported to accumulate in S-phase (43), which at least partially contributes to a reduced proliferation rate. Consistently, our immunohistochemical results also showed a lower level of Ki67 in ESCO2-depleted tumors relative to WT tumors. Collectively, these data suggest that depletion of ESCO2 inhibits tumor growth in the absence of chemotherapy agents by affecting endogenous DNA damage repair and cell cycle arrest.

The role of ESCO2 in colorectal cancer cells identified in this study provides a novel molecular explanation for CRC chemoresistance. As the main approach for the treatment of CRC, chemotherapy, including oxaliplatin, plays an important role in preoperative treatment and in reducing cancer relapse after surgery (44). However, cancer cells develop several mechanisms to evade oxaliplatin-induced cell apoptosis, one of which is the damage-induced overexpression of NHEJ repair factors and improvement of NHEJ repair efficiency, thereby resulting in drug resistance (45). The upregulation of the 53BP1 expression level promotes NHEJ repair efficiency and leads to radiotherapy resistance in colorectal cancer cells (46). Our results suggest that ESCO2 regulates the formation of 53BP1 foci. Depletion of ESCO2 in CRC cells leads to the disruption of 53BP1-MDs ring-like structure and causes cancer cells to become hypersensitive to chemotherapeutic drugs. TCGA analysis showed that the expression level of ESCO2 positively correlated with 53BP1 and BRCA1 in colorectal cancer samples (Supplementary Figure S1C). We propose that 53BP1-MDs mediated by cohesin-dependent chromatin dynamics contributes to CRC chemoresistance, and the strategies that inhibit the recruitment of ESCO2 to DBSs and/or reduce its acetyltransferase activity to disrupt the ring-like structure of 53BP1 may have therapeutic value in CRC radiotherapy and chemotherapy.

Overall, our data identify that ESCO2 plays a vital role in the DDR by regulating chromatin structure and promoting the recruitment of 53BP1. Our findings provide mechanistic insight into the relationship between the 3D genome structure and DSB repair and its role in colorectal cancer therapy.

## Supplementary Material

gkad533_Supplemental_Files

## Data Availability

All data generated are included in this article and its supplementary data files. ChIP-seq data from this study is available at the Gene Expression Omnibus (http://www.ncbi.nlm.nih.gov/projects/geo/, GSE221266). The mass spectrometry proteomics data have been deposited to the ProteomeXchange Consortium via the PRIDE [1] partner repository with the dataset identifier PXD039072 and 10.6019/PXD039072. Western blot gel images can be available at https://zenodo.org/record/7903677#.ZFcyhi-9GTc.
