# Supplementary material for: ATM–ESCO2–SMC3 axis promotes 53BP1 recruitment in response to DNA damage and safeguards genome integrity by stabilizing cohesin complex
Source: Nucleic Acids Res. 2023 Jun 28;51(14):7376–91. doi: 10.1093/nar/gkad533 (PMC10415120; doi:10.1093/nar/gkad533)
Supplement: gkad533_Supplemental_Files [file gkad533_supplemental_files.zip › supplementary information revision.pdf]

Supplemental Data

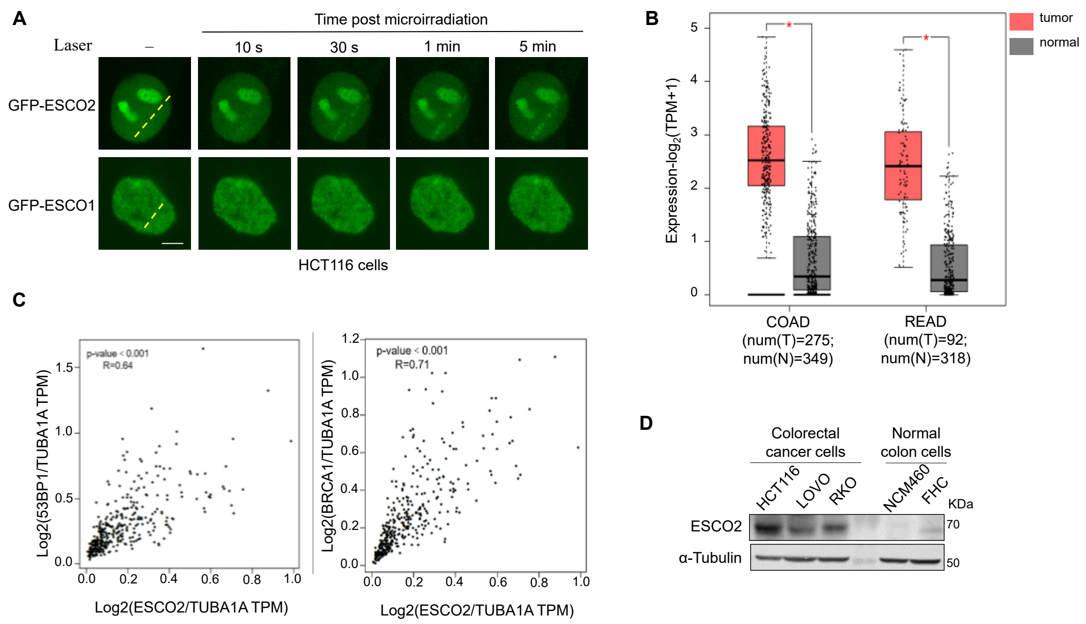

Figure S1. ESCO2 is highly expressed in colorectal cancer. (**A**) HCT116 cells transfected with GFP-ESCO2 or GFP-ESCO1 were subjected to a laser microirradiation assay. GFP fluorescence was detected by fluorescence microscopy at the indicated time points. Representative images are shown. Scale bar, 2 μm. (**B**) Bioinformatics analysis of ESCO2 mRNA expression level in normal colorectal tissue and colorectal cancer (CRC) samples using TCGA and GTEx datasets. The data are presented with a box plot. Statistical analysis was performed using a Student's *t* test. * $P < 0.05$. (**C**) Expression correlation analysis of ESCO2 with 53BP1 (left panel) and BRCA1 (right panel) in the TCGA COAD and READ data sets using GEPIA2. (**D**) Western blot analysis of ESCO2 expressions in normal colon cells and CRC cells.

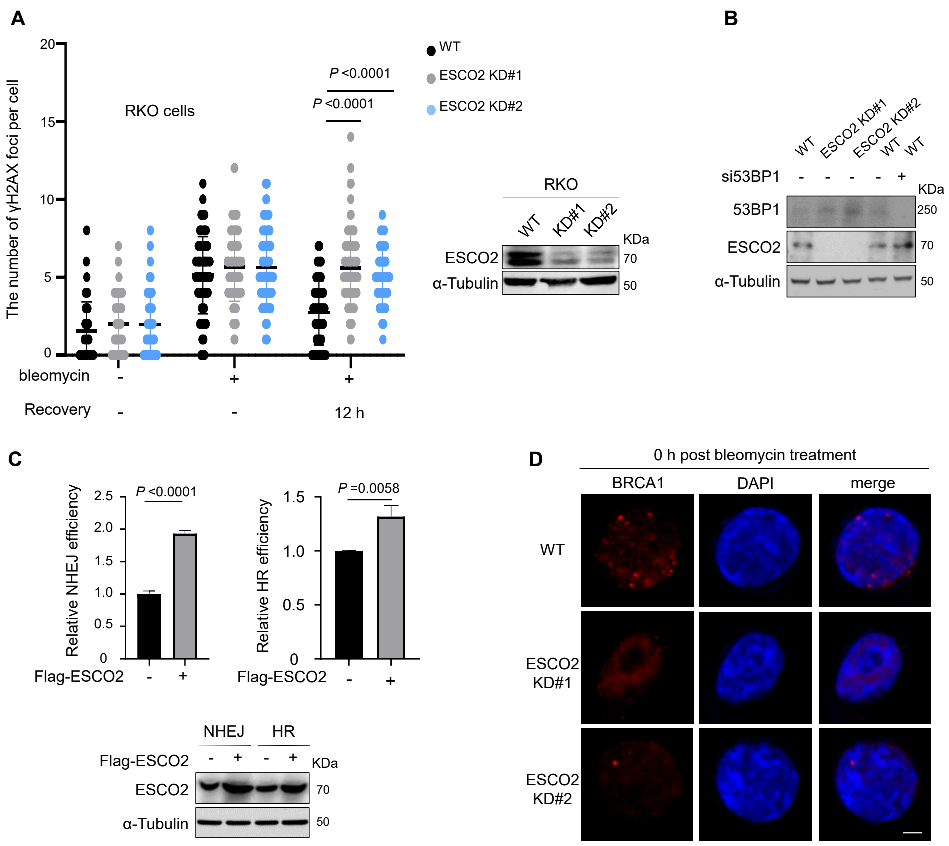

Figure S2. ESCO2 is involved in DSB repair. (**A**) Wild-type (WT) and ESCO2 knockdown RKO cells were treated with (+) or without (-) bleomycin (5 μM, 2 h), and then immunofluorescence assays were performed at the indicated time points after DNA damage to examine γH2AX foci. Representative images from three independent experiments are shown. The graph shows mean ± SEM; n = 90 for each group. Statistical analysis was performed using a Student's t test. Western blotting was conducted to assess the efficiency of ESCO2 knockdown in RKO cells. (**B**) Western blotting was conducted to assess the efficiency of 53BP1 knockdown in the siControl-transfected or siMDC1-transfected HCT116 cells that were used in the NHEJ assays. (**C**) HEK293T cells were transfected with the indicated plasmids, and the efficiency of NHEJ and HR repair was determined and presented relative to HEK293T cells transfected with Flag-ev. Statistical analysis was performed using a Student's t test. Western blotting was performed to assess the overexpression level of ESCO2 in HEK293T cells. (**D**) Wild-type (WT) and ESCO2 knockdown HCT116 cells were treated with bleomycin for 2 h and immunofluorescence assays were then performed to examine the BRCA1 foci. Scale bar, 2 μm.

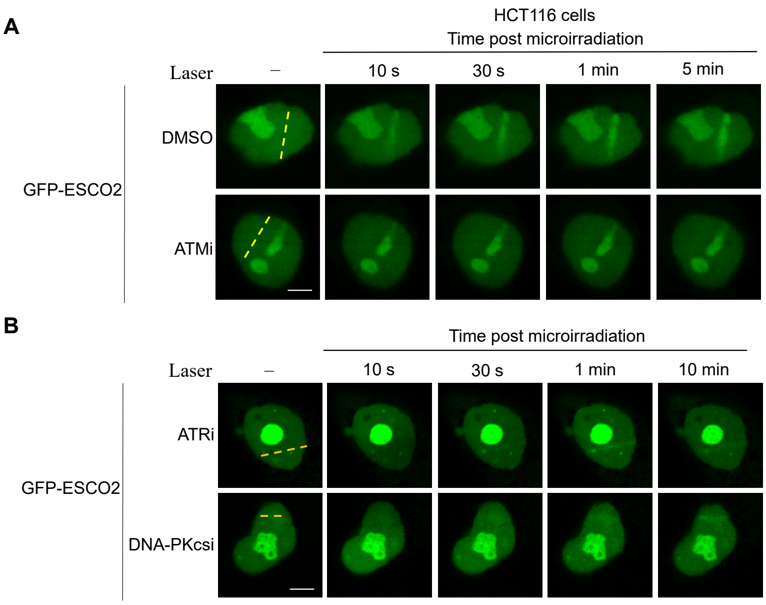

Figure S3. Screening the kinase that regulates ESCO2 recruitment. (**A**) HCT116 cells were transfected with GFP-ESCO2 for 36 h, and treated with 10 μM ATM inhibitor KU55933 for 2 h followed by laser microirradiation assays. Scale bar, 2 μm. (**B**) HeLa cells transfected with GFP-ESCO2 were treated with 2 μM DNA-PK inhibitor NU7441 or 5 μM ATR inhibitor VE821 for 2 h, after which laser microirradiation assays were performed. Scale bar, 2 μm.

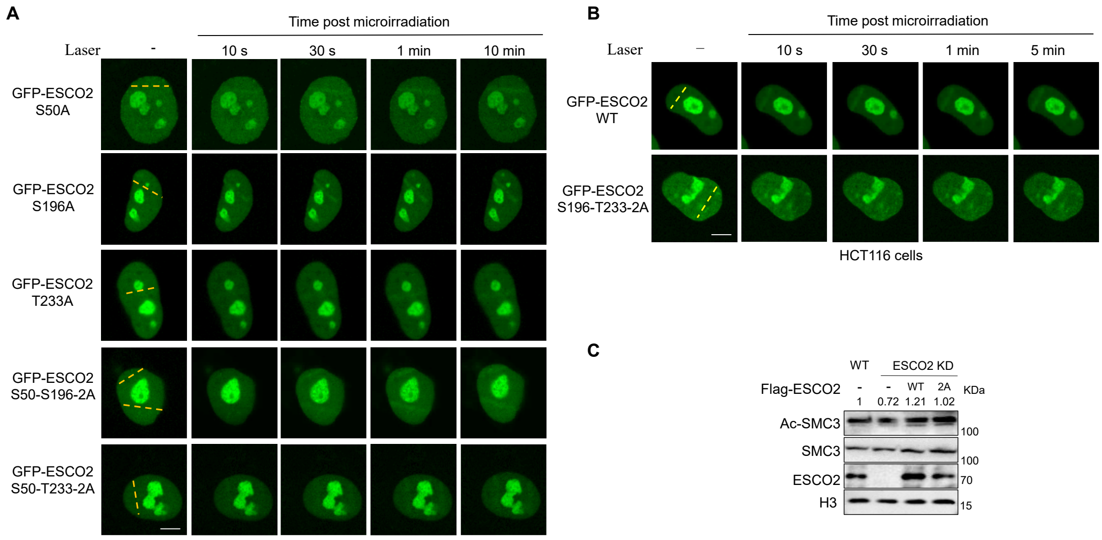

Figure S4. Screening the phosphorylation sites of ESCO2. (**A**) HeLa cells transfected with the GFP-ESCO2 mutation plasmid were subjected to a laser microirradiation assay. GFP fluorescence was detected by fluorescence microscopy at the indicated time points. Representative images are shown. Scale bar, 2 μm. (**B**) HCT116 cells transfected with GFP-ESCO2 wild-type (WT) or GFP-ESCO2 S196-T233-2A plasmid were subjected to a laser microirradiation assay. GFP fluorescence was detected by fluorescence microscopy at the indicated time points. Representative images are shown. Scale bar, 2 μm. (**C**) Western blot analysis of the acetylation level of SMC3 in WT, ESCO2 KD, and ESCO2 KD HCT116 cells transfected with the indicated plasmids.

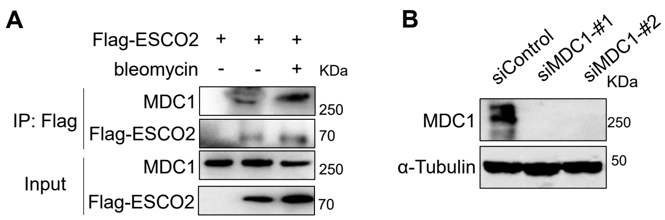

Figure S5. MDC1 interacts with ESCO2. (**A**) The interaction between MDC1 and ESCO2 was confirmed by enriching Flag-ESCO2 in HCT116 cells with (+) or without (-) bleomycin (5 μM, 2 h). (**B**) Western blotting was conducted to assess the efficiency of MDC1 knockdown in HCT116 cells transfected with siControl or siMDC1.

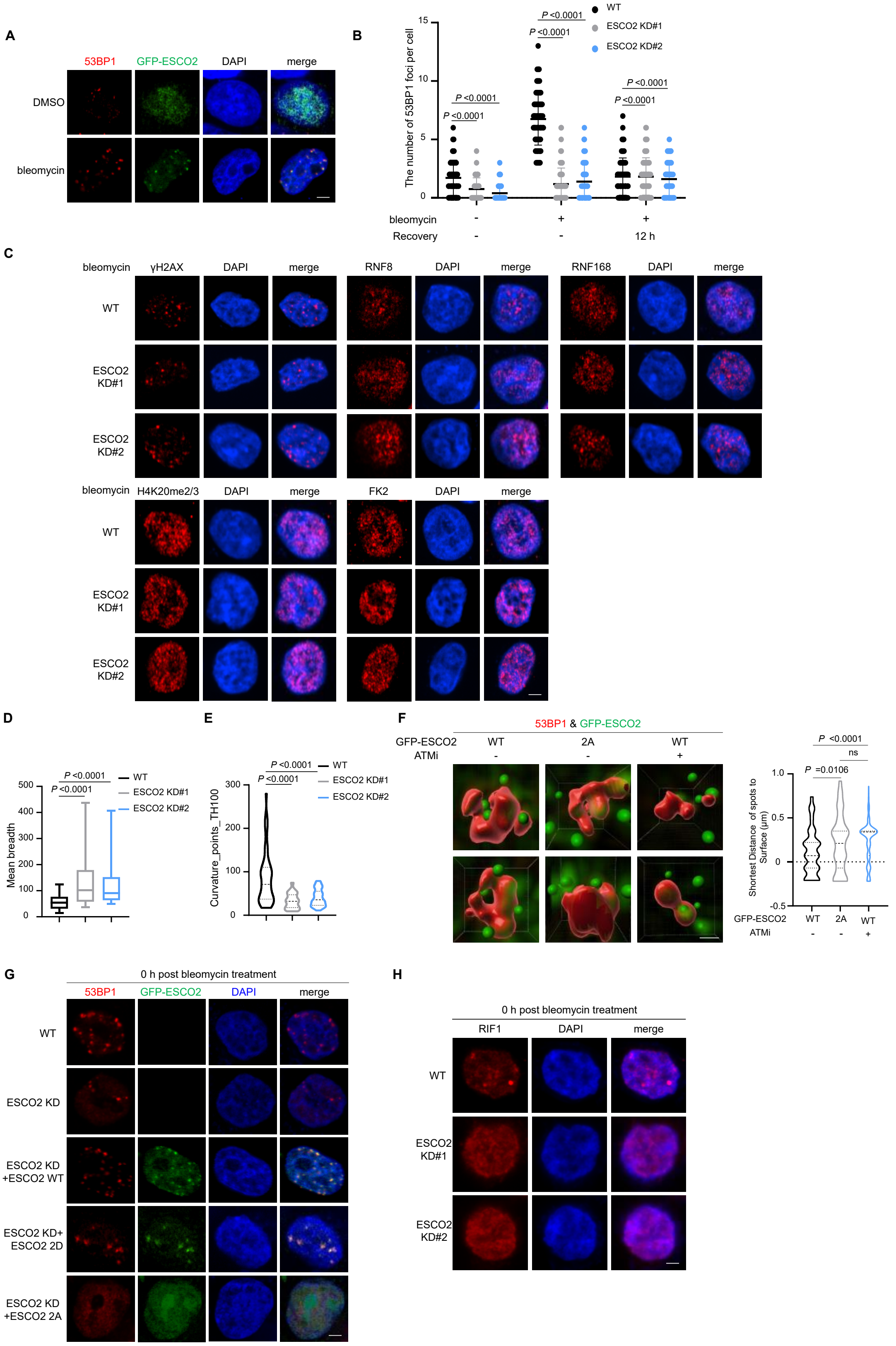

Figure S6. ESCO2 promotes the formation of 53BP1 microdomains. (**A**) HCT116 cells were treated with or without bleomycin for 2 h. Immunofluorescence assays were then performed to examine the colocalization of ESCO2 and 53BP1 foci. Scale bar, 2 μm. (**B**) Wild-type (WT) and ESCO2 knockdown RKO cells treated with (+) or without (-) bleomycin (5 μM, 2 h) were

subjected to immunofluorescence assays at the indicated time points after DNA damage. Representative images of 53BP1 foci from three independent experiments are shown. The graph shows mean ± SEM; n = 90 for each group. Statistical analysis was performed using a Student's *t* test. (**C**) ESCO2 WT and ESCO2 KD HCT116 cells were treated with bleomycin for 2 h. Immunofluorescence assays were performed to examine the γH2AX, RNF8, RNF168, H4K20me2/3, and FK2 foci. Scale bar, 2 μm. (**D**) QUANTEX analysis of mean-breadth of 53BP1 in WT and ESCO2 knockdown RKO cells. The graph shows mean ± SEM; n = 50 for each group. Statistical analysis was performed using a Student's *t* test. (**E**) QUANTEX analysis of Curvature_point_TH100 of 53BP1 in WT and ESCO2 knockdown RKO cells. The graph shows mean ± SEM; n = 50 for each group. Statistical analysis was performed using a Student's *t* test. (**F**) Three-dimensional reconstruction was performed for 53BP1 and GFP-ESCO2 fluorescence images in the indicated treatment, and the distance from ESCO2 to the 53BP1-MD surface was calculated. Statistical analysis was performed using a Student's *t* test. (**G**) HCT116 cells transfected with the indicated plasmid were treated with bleomycin for 2 h. Immunofluorescence assays were then performed to examine the 53BP1 foci. Scale bar, 2 μm. (**H**) WT and ESCO2 knockdown HCT116 cells were treated with bleomycin for 2 h, and then the RIF1 foci were examined by immunofluorescence assays. Scale bar, 2 μm.

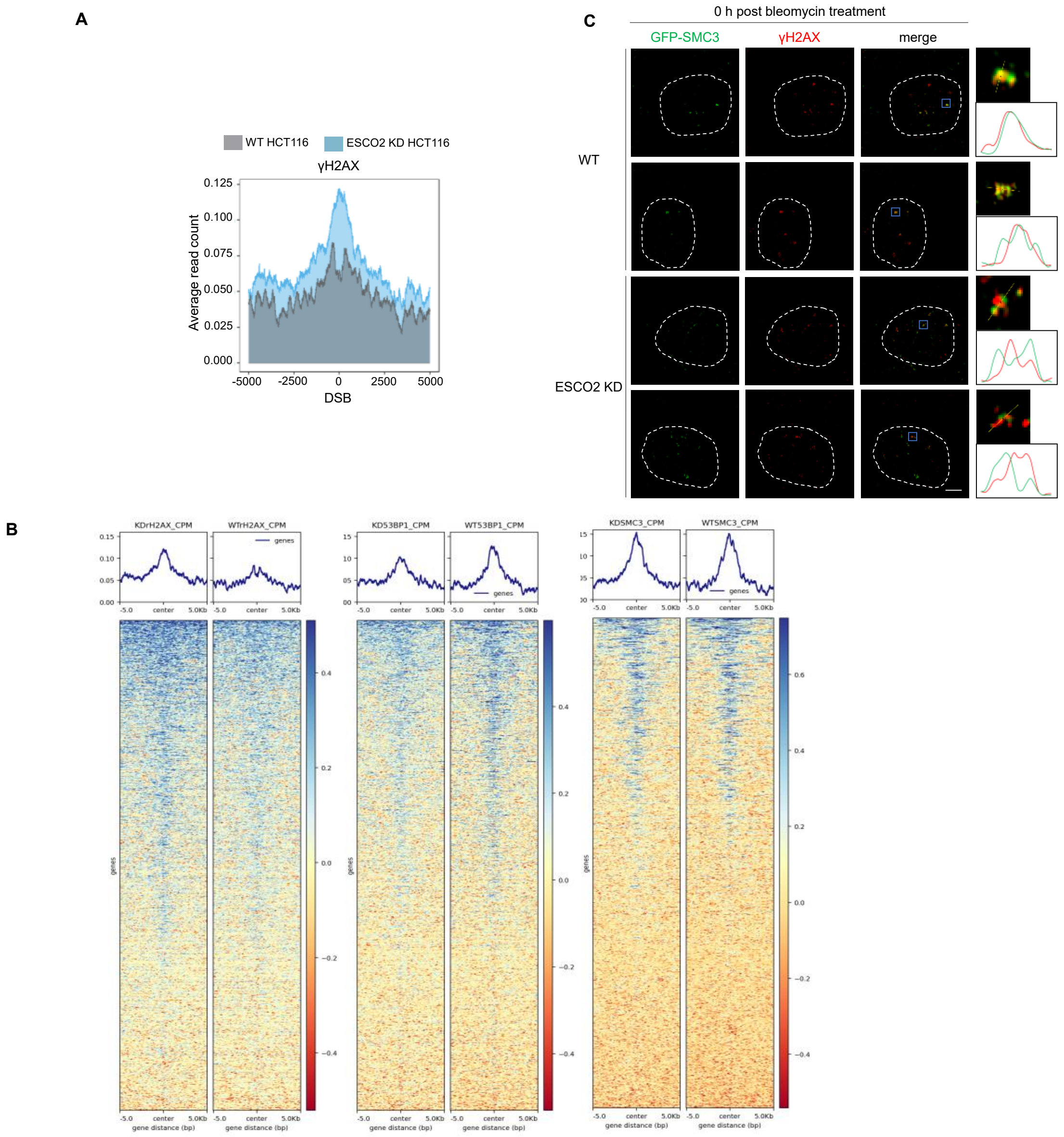

Figure S7. ESCO2 promotes the formation of 53BP1 foci by acetylating SMC3 K105/106. (**A**) Average profile for γH2AX in ESCO2 WT and ESCO2-depleted HCT116 cells. ChIP-seq analyses of WT and ESCO2-depleted HCT116 cells after 4-OHT treatment (500 nM, 4 h), using anti-γH2AX antibodies. Averaged γH2AX signals over a 10-kb region flanking annotated AsiSI sites are shown. (**B**) Heatmap representing the γH2AX, 53BP1 and ac-SMC3 ChIP-seq signals on a 10 kb window centered around all AsiSI sites, ordered based on the mean value per region. (**C**) ESCO2 WT and ESCO2 KD HCT116 cells were treated with bleomycin (5 μM, 2 h), and immunofluorescence assays were then performed to examine the co-localization of SMC3 and γH2AX foci by SIM super-resolution microscopy. For each row of images, the bottom right panel shows the distribution of the red and green signals along the white dashed line from the top right image, which is indicated by the blue box in the merge images. Scale bar, 2 μm.

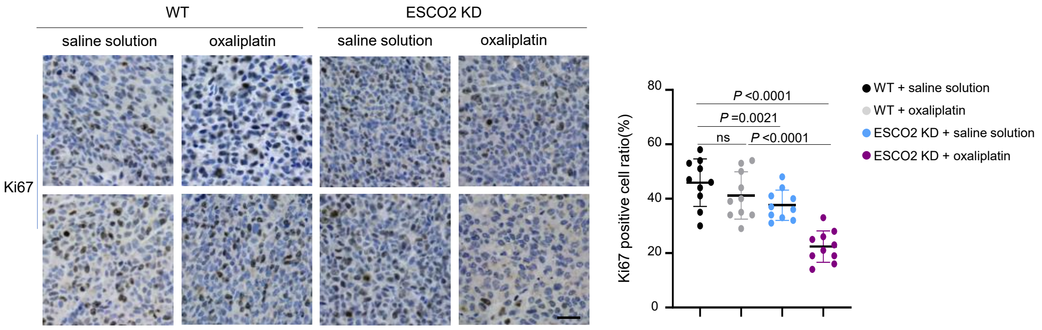

Figure S8. ESCO2 depletion decreases the level of Ki67 in xenograft tumors. The levels of Ki67 in the xenograft tumors were immunohistochemically evaluated. Scale bar, 20 μm. The graphs show mean ± SEM.

Supplementary Table S1. ChIP-qPCR primers

Primer name	sequence
AsiSI site1-F	CCTTCTTTCCCAGTGGTTCA
AsiSI site1-R	GTGGTCTGACCCAGAGTGGT
AsiSI site2-F	ATGCCATGTGTCCTGATGAA
AsiSI site2-R	CTGACTGGTGGCTTTTCCAT
AsiSI site3-F	GATTGGCTATGGGTGTGGAC
AsiSI site3-R	CATCCTTGCAAACCAGTCCT
AsiSI site4-F	CCCTGGAGGTAGGTCTGGT
AsiSI site4-R	CGCACACTCACTGGTTCCT
AsiSI site5-F	TGCCGGTCTCCTAGAAGTTG
AsiSI site5-R	GCGCTTGATTTCCCTGAGT
