# Supplementary material for: ATM–ESCO2–SMC3 axis promotes 53BP1 recruitment in response to DNA damage and safeguards genome integrity by stabilizing cohesin complex
Source: Nucleic Acids Res. 2023 Jun 28;51(14):7376–91. doi: 10.1093/nar/gkad533 (PMC10415120; doi:10.1093/nar/gkad533)
Supplement: gkad533_Supplemental_Files [file gkad533_supplemental_files.zip › Supplementary Table S2-3 legends.docx]

**Supplementary Table S2.** HEK293T cells transfected with Flag-ESCO2 were treated with bleomycin and then Flag-ESCO2 was immunopurified using anti-Flag antibodies and subjected to mass spectrometric analysis. The IP-MS results are listed in the table.

**Supplementary Table S3.** ChIP assays were performed in AsiSI–ER-HCT116 cells after 4 h 4-OHT treatment, using anti-53BP1 and anti-ac-SMC3 antibodies. And qRT-PCR was performed to assess ac-SMC3 and 53BP1 enrichment using input DNA, ChIP enriched DNA, and primers (listed in Supplementary Table S1). Samples were heated to 95 °C for 5 min and then amplified for 40 cycles at 95 °C for 30 s, 60 °C for 30 s and 72 °C for 30 s. The Ct values were listed in the table.
